# Armored BCMA CAR T Cells Eliminate Multiple Myeloma and Are Resistant to the Suppressive Effects of TGF-β

**DOI:** 10.3389/fimmu.2022.832645

**Published:** 2022-02-09

**Authors:** Leah M. Alabanza, Ying Xiong, Bang Vu, Brian Webster, Darong Wu, Peirong Hu, Zhongyu Zhu, Boro Dropulic, Pradyot Dash, Dina Schneider

**Affiliations:** Research and Development, Lentigen, a Miltenyi Biotec Company, Gaithersburg, MD, United States

**Keywords:** CAR T cells, multiple myeloma, cell therapy, lentiviral (LV) vector, TGF - β1

## Abstract

CAR T-cell therapies targeting the B-cell maturation antigen eliminate tumors in relapsed/refractory multiple myeloma patients, however durable remissions remain difficult to attain. Transforming growth factor beta (TGF-β) is a multifunctional cytokine abundantly expressed in the multiple myeloma bone marrow niche, where it promotes an immunosuppressive tumor microenvironment. We hypothesized that BCMA CAR T-cells armored to resist the suppressive effects of TGF-β will provide an advantage in treating multiple myeloma. The armored B2ARM CAR T cells, co-expressing BCMA targeting CAR with TGF-β dominant-negative receptor II, were generated by lentiviral transduction of primary human CD4+ and CD8+ T cells. The B2ARM CAR T cells eliminated MM.1S multiple myeloma targets in long-term cytotoxicity assays, even under TGF-β-high conditions, whereas cytotoxic function of the non-armored B2 CAR -T cells was inhibited by TGF-β. Concordantly, after long-term exposure to targets in the presence of TGF-β, the B2ARM CAR T cells were enriched for Granzyme B, CD107a, Ki67 and polyfunctional cells T-cells (double or triple-positive for IFN-γ, IL-2 and/or TNF-α), as determined by flow cytometry. In addition, the B2ARM CAR T-cells, but not the conventional B2 CAR T-cells, resisted the TGF-β-mediated suppression of activation (CD25), exhaustion (PD-1, LAG3), and differentiation to T effectors (CD45RA+ CD45RO-CD62L-). In NSG mice bearing RPMI-8226 tumors overexpressing TGF-β, the B2ARM CAR mediated 100% tumor rejection and survival, superior infiltration of tumors on day 7 post CAR T treatment (%CD3+CAR+), and greater expression of IFN-γ, TNF-α, Ki67, Granzyme B, and PD-1, as compared to tumor-infiltrating non-armored B2 CAR T-cells. In NSG RPMI-8226 xenograft model in which tumors were additionally supplemented with TGF-β injections on days -1 through 11 of CAR T treatment, the B2ARM CAR T cells rejected tumors faster than the non-armored B2 CARs, and showed greater numbers of CD3+ and CD3+CAR+, central memory (CD45RO+CD62L+) and effector memory (CD45RO+CD62L-) T cells in the peripheral blood 18 days after treatment. In summary, the armored B2ARM CAR T cells mediate superior persistence, proliferation, multi-functionality, effector differentiation and anti-tumor function in pre-clinical models of multiple myeloma, while abrogating TGF-β-mediated suppression.

## Introduction

Despite the recent surge in the development of novel therapeutics, multiple myeloma (MM), a hematologic disorder characterized by malignant proliferation of plasma cells, remains incurable. The advent of immunotherapies, including CAR T-cell therapy, has improved the prognosis of patients with relapsed/refractory multiple myeloma (RRMM) (1). However, while CAR T-cell therapy has resulted in high overall response rates in patients with RRMM, the durability of the responses has been limited (1–3). Most clinical studies to date have reported relapses and/or non-responders. A number of relapses after treatment with B-cell maturation antigen (BCMA) CAR T-cells have been attributed to the downregulation of BCMA on MM cells and/or the emergence of BCMA negative MM clones (4–7). However, several clinical trials have also observed non-responders and relapses despite the persistent expression of BCMA on MM cells (4, 8, 9), an indication that failure with CAR T-cell therapy may also potentially occur as a result of insufficient expansion, lack of persistence, or loss of efficacy of CAR T-cells.

MM cells predominantly localize in the bone marrow (BM), and the development and accumulation of MM in the BM is supported by a BM microenvironment replete in factors that specifically promote the expansion of MM cells and facilitate escape from immunosurveillance (10–12). Of particular importance in this context is transforming growth factor beta (TGF-β), a multifunctional cytokine known to regulate various cellular functions, including cell proliferation, differentiation, and immunosuppression (13, 14). In the BM microenvironment, the latent form of TGF-β is generated by a number of sources, including BM stromal cells, osteoblasts, osteoclasts, as well as MM cells, resulting in the abundant deposition of TGF-β in bone matrices and in the BM milieu (15, 16). Furthermore, MM cells are known to enhance osteoclastic bone resorption, which not only gives rise to the formation of bone lesions in MM, but also results in the release and activation of TGF-β from bone matrices (17). Latent TGF-β in the BM microenvironment can be additionally activated by acids and metalloproteinases produced by osteoclasts (18, 19), thus further enriching the BM milieu with activated TGF-β. In turn, activated TGF-β is a contributing factor in the propagation of MM by inhibiting the differentiation of immature mesenchymal stromal cells into mature osteoblasts, which not only contributes to further bone destruction in MM but results in the growth and survival of MM cells (15, 20, 21). Furthermore, the presence of TGF-β in the BM microenvironment also results in the production of various soluble factors that additionally foster MM expansion. Specifically, TGF-β induces both MM cells and stromal cells to produce IL-6, which has been shown to mediate MM cell growth (16). Both IL-6 and TGF-β, in turn, are cytokines that facilitate the differentiation of T helper 17 (TH17) cells, resulting in the elevation of IL-17 in the BM milieu, which further promote the expansion of MM cells (22).

In addition to supporting the tumor expansion of MM cells, TGF-β may contribute to the immunosuppressive conditions in the BM milieu, allowing MM cells to evade the immune response. Specifically, TGF-β is notable for being a potent T-cell suppressor. Its regulation of T-cell function is predominantly mediated through its binding to the receptor TGF-βRII, which forms a tetrameric receptor with TGF-βRI, consequently resulting in the phosphorylation of downstream signaling molecules (23). Through this mechanism, TGF-β inhibits T-cell functionality on various fronts, including by limiting the proliferative capacity of T-cells. TGF-β suppresses T-cell proliferation by mediating the expression of cell cycle regulators, such as c-Myc, p21^Cip1^, and p27^Kip1^, as well as through downregulation of IL-2 production from T-cells (24–26). In congruence to this, mechanistic studies *in vitro* have shown that TGF-β secreted by MM cells inhibited T-cell proliferation and T-cell responsiveness to IL-2 (27). TGF-β is also suppressive to the cytotoxic functions of CD8+ T-cells, specifically by repressing granzyme B and IFN-γ production (28). T-cell differentiation, likewise, can be inhibited by TGF-β signaling *via* silencing the expression of transcription factors, T-BET and GATA-3, and resulting in the abrogation of Th1 and Th2 differentiation, respectively, in CD4+ T-cells (29–31). Additionally, TGF-β is known to induce the T-reg phenotype in naïve CD4+ T-cells by eliciting the expression of the transcription factor, FOXP3; thus, in addition to directly suppressing T-cell functionality, TGF-β can generate the T-reg subset, which further suppress the effector functions of T-cells (32, 33).

A number of studies have noted elevated levels of TGF-β in the BM and peripheral blood of MM patients (34–37). Several studies have additionally reported that there is a discernable impairment in the functionality of immune cells from MM patients, including T-cell dysfunction (38–40). Furthermore, TGF-β has been directly implicated in instances of immune dysregulation, such as the defective upregulation of CD80 on dendritic cells from the peripheral blood of MM patients (41) and the impaired proliferation of T-cells as a result of TGF-β produced by either MM cells or by BM mesenchymal stromal cells derived from MM patients (27, 42). While the reasons for the lack of CAR T-cell expansion and persistence that have been observed in non-responder and relapsed MM patients are not yet well delineated, the elevated levels of TGF-β in the peripheral blood and BM of MM patients may potentially be a contributing factor. Therefore, to counteract the suppressive conditions of the TGF-β-enriched BM microenvironment, we have designed a novel BCMA CAR that co-expresses a dominant-negative (DN) TGFBRII, which lacks the kinase domain of the wild type receptor. The DN TGFBRII is still functionally capable of binding TGF-β and forming a tetrameric receptor complex with TGFβRI, but it lacks the kinase domain needed to mediate downstream signaling of TGF-β (43). The DN TGFBRII, therefore, endows the BCMA CAR T-cells with resistance to the inhibitory effects of TGF-β.

In this study, we show that the BCMA CAR with DN TGFBRII, termed B2ARM, maintains high functionality, both *in vitro* and *in vivo*, despite prolonged exposure to TGF-β. This novel BCMA CAR design may rectify the lack of durable effectiveness that remains to be the major impediment of BCMA CARs currently in the clinic.

## Results

### A BCMA CAR With a Fully Human Single Chain Variable Fragment Exhibits Potent Anti-Tumor Activity

**Figure 1:**
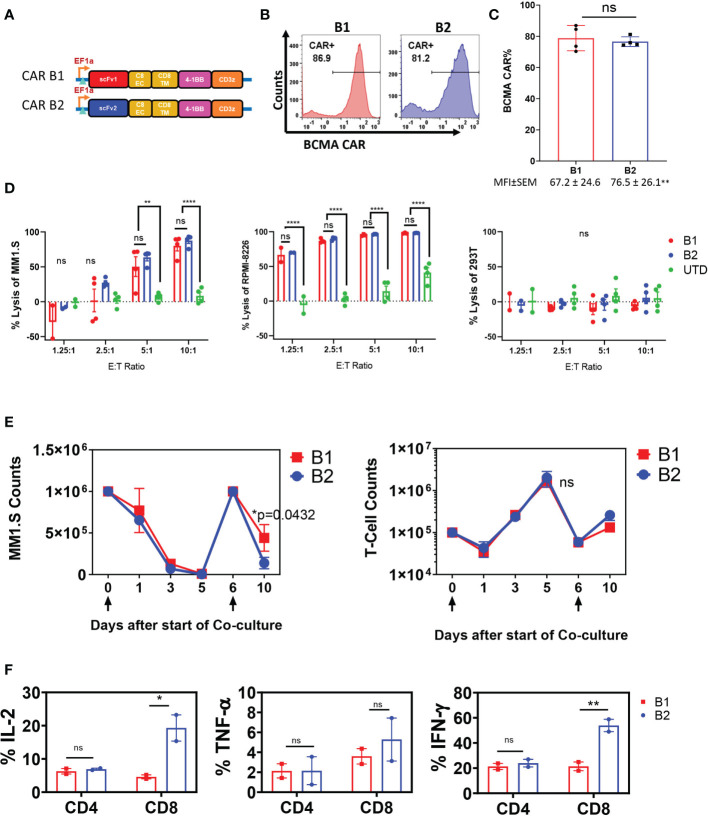
Characterization of B1 and B2 CAR functionality *in vitro*. **(A)** CARs B1 and B2 are comprised of fully human scFv linked to the human CD8 extracellular and transmembrane domains, followed by the 4-1BB co-stimulatory domain, and the CD3ζ activation domain. **(B)** Representative flow diagrams of CAR T expression from one donor. **(C)** Mean ± SEM CAR expression from four transduction experiments performed on T cells from different healthy donors are plotted as bars, and the corresponding mean fluorescence intensity values (MFI) are noted below each bar. Statistical significance was determined by Student t-test, ns- non-significant. Transduction at MOI 10 for B2 and MOI 40 for B1 was used in order to achieve similar CAR expression. **(D)** Overnight killing assay on BCMA-positive MM lines MM.1S and RPMI-8226, and BCMA-negative line 293T. The cytotoxic capacity of B1 and B2 CAR T-cells were determined by co-culturing the CAR T-cells with target cells for 18-20 hours at the ETT ratios shown. The percent cytotoxicity was determined based on the luciferase activity of the remaining target cells in the co-culture after incubation with the CAR T-cells. Mean +/- SEM of four separate experiments with T cells from different donors, each performed in triplicate, are shown. Statistical significance was determined by two way ANOVA with Tukey’s multiple comparisons *post-hoc* test; ns-non significant, **p<0.01, ****p<0.001. **(E)** Long-term co-incubation study with BCMA CAR T cells and MM.1S-GFP target cells. Data are presented as mean+/- SEM of CAR T cells from two separate donors tested in long-term co-incubation experiments. Target cells at ETT ratio of 0.1 were added on days 0 and 6, as indicated by arrows below the x-axis. The absolute counts of T cells and target cells were assessed by flow cytometry at the indicated time points by quantifying the number of CD3+ cells and GFP+ cells, respectively. Statistical significance was determined by two way ANOVA with Tukey’s multiple comparisons *post-hoc* test. **(F)** Elaboration of cytokines IL-2, TNF-α, and IFN-γ by CAR T cells on day 11 of the long-term co-culture experiment was determined by intracellular staining of in CD3+ cells and flow cytometric analysis. Data represent mean +/-SEM of two separate long-term experiments, using CAR T cells from different healthy donors. Statistical significance was determined by two way ANOVA with Sidak’s multiple comparisons *post-hoc* test; *p<0.05, **p<0.01, ns, non-significant.

**Figure 2:**
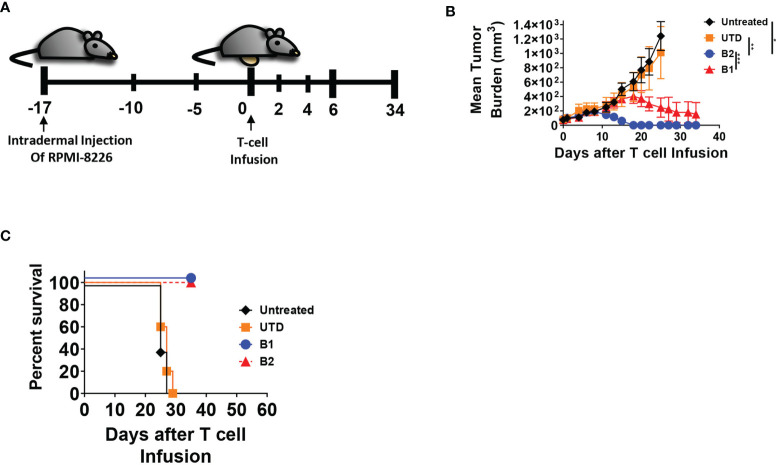
B2 CAR T-cells are more potent in eradicating tumors *in vivo* compared to B1 CAR. **(A)** In the *in vivo* tumor model, 8 x 10^6^ RPMI-8226 cells were intradermally injected on the abdomen of NSG mice (all groups n = 8, except untreated, n = 5) and allowed to engraft for 17 days before the intravenous injection of T-cells. Five million CAR T-positive -cells was infused per mouse, normalized based on CAR expression, or equivalent amount of non-transduced T cells (UTD), or left untreated. Mice were monitored for **(B)** tumor growth and **(C)** survival. Statistical significance was determined by paired Student t-test for the indicated groups, *p<0.05, **p<0.01, ***p<0.001.

The targeting domains of BCMA CARs B1 and B2 were constructed with two distinct fully human single chain fragment variable (scFv) sequences (**Supplementary Figure 1A**). The sequences were derived from a human yeast display scFv library directed toward the full-length human BCMA ectodomain. Each BCMA CAR was comprised of the scFv targeting domain followed by CD8 extracellular and transmembrane domain, and the cytoplasmic 4-1BB and CD3ζ domains (**Figure 1A**). Due to differences in transduction efficiency between CARs B1 and B2, and to achieve similar expression in human primary T cells, we transduced CAR B1 at multiplicity of infection (MOI) 40, and CAR B2 at MOI 10 (**Figure 1B**), and reproducibly achieved similar percentage of ~80% CAR expression in T cells from four different healthy donors, whereas the MFI was somewhat higher in B2 CAR transductions, despite the lower MOI of lentiviral vector used (p<0.01), (**Figure 1C**). The functionality of CARs B1 and B2 was evaluated in an overnight cytotoxicity assay against two multiple myeloma (MM) cell lines, MM.1S and RPMI-8226, both positive for BCMA expression (**Figure 1D**, **Supplementary Figure 1B**). CARs B1 and B2 consistently exhibited equipotent and high cytotoxicity against MM.1S and RPMI-8226 target cells after overnight co-culture with the target cells at multiple effector to target (ETT) ratios, p<0.01-p<0.0001). No killing of the BCMA-negative line 293T was detected with either B1 or B2 CAR (**Figure 1D**), demonstrating CAR specificity. We further examined the phenotypic and functional characteristics of BCMA CARs B1 and B2 during long-term exposure to target cells through consecutive spiking with MM.1S cells on days 0 and 6 (**Figure 1E**). Both CARs B1 and B2 exhibited similar ability to eradicate the tumor cells during initial days of co-culture; however, B1 CAR was less potent than B2 CAR at the end of the co-culture period (p=0.0432, **Figure 1E**). We observed that B2 CAR retained higher CAR percentage throughout the long-term exposure to antigens compared to B1 (**Supplementary Figure 2A**), and exhibited a higher activation status based on the induction of cluster of differentiation CD25 (p=0.032, **Supplementary Figure 2B**), whereas the differences in the induction of CD69 were not statistically significant (**Supplementary Figure 2C**), during long-term co-culture exposure to target cells. Furthermore, the cytokine production of the CAR T-cells on day 11 of the long-term co-culture shows higher production of IL-2 and IFN-γ cytokines from the B2 CD3+CD8+ T-cell subsets compared to the B1 T-cells, whereas the difference in the induction of TNF-α was not statistically significant (**Figure 1F**). These results show B2 CAR’s capability to retain its cytokine production and cytotoxic function despite prolonged exposure to target antigens. We next evaluated the ability of the B1 and B2 CARs to eradicate tumors in an *in vivo* intradermal tumor model with RPMI-8226 cells, in which tumor bearing mice were treated with 5 x 10^6^ CAR T-positive cells, or UTD control, or left untreated (**Figure 2A**). The tumor sizes for all the mice that received the B1 or B2 CAR T-cells decreased (**Figure 2B**), and both B1 and B2 CARs mediated 100% survival in this model (**Figure 2C**). However, the B2 CAR T-cells mediated a greater and more rapid regression as compared to B1 CAR (p<0.001), completely rejecting the tumors in all mice by day 18 after CAR T-cell infusion, while treatment with the B1 CAR T-cells resulted in a slower decline of tumor sizes, was not significantly different from the control groups Untreated and UTD, and the tumors were not completely eradicated in 3 out of the 5 mice by study end (**Figure 2B**). Overall, the B2 CAR showed superior efficacy compared to the B1 CAR in controlling and eradicating tumors in the *in vivo* MM tumor model.

### Co-Expression of the Dominant Negative TGFBRII Element With the B2 CAR Results in Resistance to the Suppressive Effects of TGF-β

**Figure 3:**
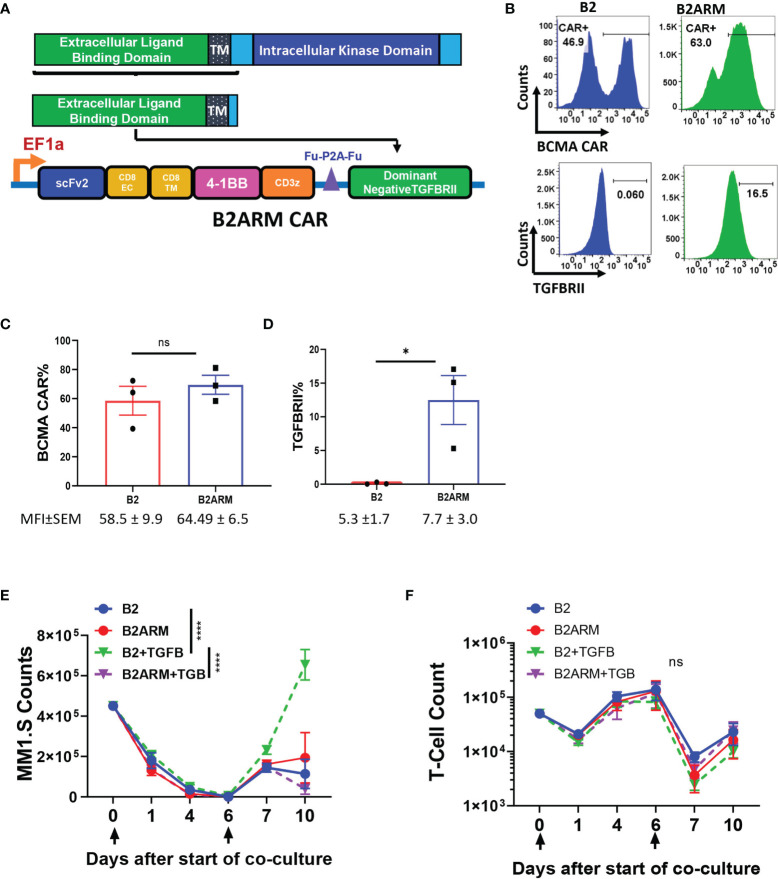
The B2ARM T-cells with the truncated TGFBRII dominant negative receptor are resistant to the suppressive effects of TGF-β. **(A)** To create the armored B2ARM BCMA CAR, the sequence of the extracellular and transmembrane domains of TGFBRII, excluding the intracellular kinase domain, was cloned in frame downstream of the B2 CAR construct. **(B)** T-cells were transduced with lentiviral vectors containing either the B2 CAR construct at MOI 10, or the B2ARM construct at MOI 80, in order to compensate for the lower transduction efficiency of the armored B2ARM CAR construct, which is larger. The cell surface expression of the BCMA CAR (upper panel) and TGFBRII (lower panel) was assessed by flow cytometry. One representative donor is shown. **(C)** Pooled results from transduction experiments with B2 and B2ARM constructs showing the expression of **(C)** the CAR and **(D)** the TGFBRII armor in T cells from three separate donors, mean ± SEM. *p<0.05, Student t-test, ns, non-significant. Mean ± SEM of mean fluorescence intensity (MFI) values for each experimental group are shown below the figures. In the long term co-culture experiment, CAR T-cells were co-incubated with the target cells, MM.1S-GFP, at an ETT ratio of 0.1, and the culture was treated with 10ng/ml of TGF-beta or remained untreated. When less than 15% of target cells remained on day 6, the co-culture was extended for a second round by adding the co-culture cells from the previous round to fresh target cells. The absolute counts of **(E)** T-cells and **(F)** target cells at different time points during the long-term co-culture was assessed by quantifying the number of CD3+ and GFP+ cells *via* flow cytometry using absolute counting beads. Data represent mean ± SEM of separate long term experiments performed in T cells from three different donors. Statistical analysis was performed by two way ANOVA with Tukey’s multiple comparisons test, ****p<0.0001.

TGF-β is a T-cell suppressive factor known to be elevated in the peripheral blood and the bone marrow (BM) of MM patients. To create an armored BCMA CAR that would remain resistant to the inhibitory effects of TGF-β, we co-expressed the B2 CAR construct with the dominant negative (DN) TGFBRII armor, that lacks the kinase domain of the receptor (**Figure 3A**), and, therefore, is incapable of mediating the suppressive signaling of TGF-β (44). We confirmed the co-expression of both the BCMA CAR and the armor by flow cytometry (**Figures 3B–D**). To adjust for differences in transduction efficiency, CAR B2 was transduced at MOI 10, and CAR B2ARM, which is larger, at MOI 80. Both the CAR and the armor, TGFBRII, were readily detected in the transduced T cells (**Figure 2B**). Since T cells also natively express TGFBRII, the overexpression of the truncated TGFBRII form in construct B2ARM was detected as a rightward population shift on the flow cytometric plot, in comparison to the non-armored B2 construct (**Figure 2B**). The CAR component of the non-armored and the armored constructs, B2 and B2ARM, respectively, were reproducibly expressed at similar percentages across transduction experiments with the adjusted MOI (**Figure 3C**), whereas reproducible overexpression of the armored truncated TGFBRII component was also confirmed by flow cytometric analysis in the B2ARM CAR T cells (**Figure 3D**).

MM cells localize in the BM where there are a number of potential sources of TGF-β, including BM stromal cells and the MM cells (27, 42). We assessed if the MM.1S and the RPMI-8226 cell lines can produce TGF-β in culture. We observed that MM.1S do not generate TGF-β in the supernatant in either latent or active form, as indicated by lack of TGF-β detection in non-acidified or acidified culture supernatants after three days of MM.1S culture (**Supplementary Figure 3A**). In order to assess the ability of the armor to resist suppression by TGF-β, 10 ng/ml TGF-β were spiked into co-cultures of armored B2ARM CAR T cells or non-armored B2 CAR T cells with MM.1S target cells, during long -term co-incubation assays (**Figures 3E, F**). Cells co-incubated without TGF-β served as a negative control. We observed a diminished capacity of the non-armored CAR B2 to clear MM.1S targets at the end of the long-term study in the presence of TGF-β, as compared to B2 CAR alone (p<0.001). However, The armored CAR B2ARM was able to completely restore CAR killing capacity in the presence of TGFB, as compared to B2+TGFB group (p<0.001, **Figure 3E**). No significant differences in CAR expansion were seen between the experimental groups, although the expansion of group B2+TGFB was the lowest in this set at the end of the co-incubation period (**Figure 3F**). These data further showcase the capacity of the armored B2ARM CAR T-cells, but not the B2 CAR T-cells, to resist the effects of the endogenous TGF-β produced by the tumor cells.

### The Armored BCMA CAR With the Dominant Negative TGFBRII Exhibits Functional Persistence Even After Long-Term Exposure to TGF-β

**Figure 4:**
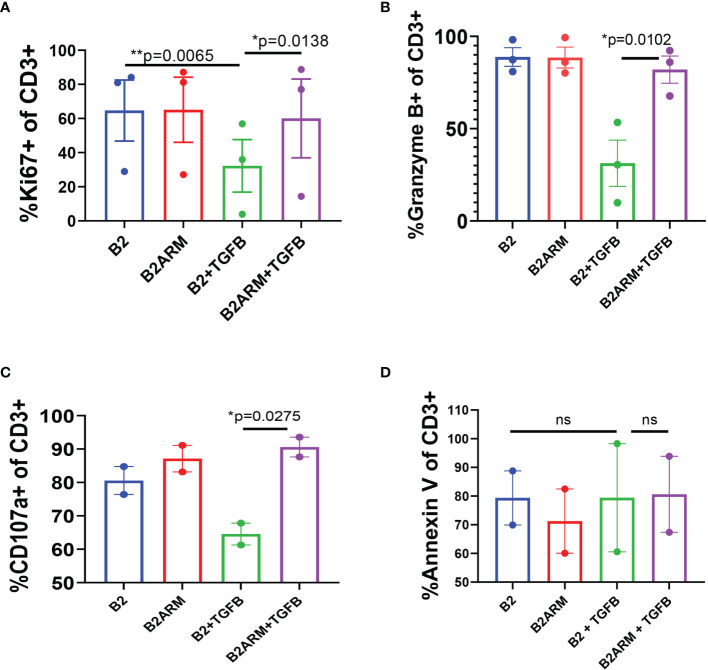
The armored B2ARM CAR maintains normal proliferative and degranulation capacity following long-term exposure to TGF-β, in contract to the non-armored CAR B2. Long-term co-cultures with target cells, MM.1S-GFP was achieved by repeatedly restimulating the co-cultured CAR T cells with target cells at ETT ratio of 0.1. Percentage expression of proliferation marker Ki67 **(A)**, degranulation markers granzyme B and CD107a **(B, C)**, respectively, as well as the apoptotic marker annexin V **(D)**, by CAR T cells were evaluated by flow cytometry at the end of the long-term co-cultures (days 8-10). Total CD3+ cells were acquired by flow cytometry for analysis. Results represent mean ± SEM from 2 or 3 separate donors. Statistical significance was determined by one way ANOVA with Tukey’s *post-hoc* test.*p<0.05, **p<0.01, ns-non-specific.

We further examined the phenotypic characteristics of the B2ARM and B2 CAR T-cells following long-term exposure to target cells in the presence of TGF-β. We evaluated CAR proliferation capacity, degranulation status, and apoptotic potential in TGF-β-exposed long term co-cultures of B2 and B2ARM CARs, by assessing the expression of the specific markers Ki67, Granzyme B, CD107a, and annexin V, respectively, at the end of the long term co-culture with MM.1S cells (**Figure 4**). Flow cytometric analysis was performed on culture days 8-10, at the end of co-culture with MM.1S target cells in the absence or presence of 10 ng/ml TGF-β, and results were pooled from 2-3 independent experiments with T cells from separate donors.

The percentage of B2 T cells expressing Ki67 was significantly decreased in the presence of TGF-β, as compared to B2 in the absence of TGF-β (p=0.0065, **Figure 4A**). However, the expression of Ki67 in B2ARM T-cells treated with TGF-β remained high and similar to the untreated CAR T B2 (p=0.0138, **Figure 4A**), indicating that the proliferation capacity of the B2ARM CAR T-cells was not inhibited by TGF-β. Similarly, the expression of degranulation markers Granzyme B and CD107a was diminished in B2 non-armored CAR T cell group in the presence of TGF-β, but remained unperturbed in the armored B2ARM group treated with TGF-β (p=0.0102, **Figure 4B** for Granzyme B; p=0.0275, **Figure 4C** for CD107a). Since TGF-β also regulates cell apoptosis, we monitored the expression of the apoptotic marker annexin V on T cells in the different treatment groups (**Figure 4D**). In contrast to Ki67, Granzyme B and CD107a, there were no differences in annexin V binding between B2ARM and B2 CAR T-cells in this assay after long-term exposure to TGF-β (**Figure 4D**). Taken together, these findings further illustrate the ability of the B2ARM T cells to resist the TGF-β - mediated suppression of T cell effector function.

**Figure 5:**
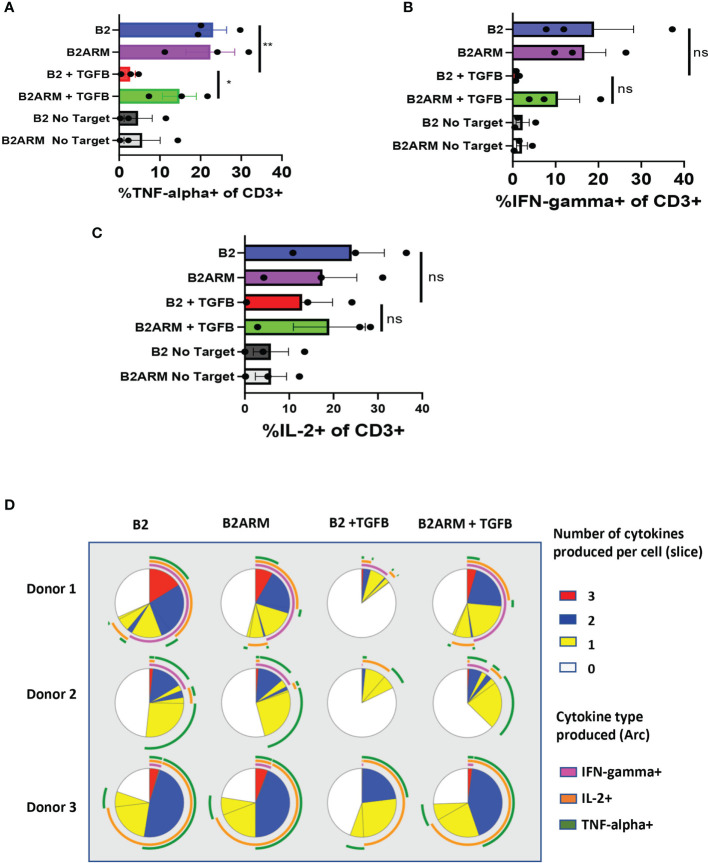
The B2ARM CAR attenuates the suppression of cytokine responses and maintains polyfunctionality after prolonged exposure to TGF-β. Cytokine production in CD3+ cells was determined by intracellular staining and flow cytometry analysis on day 8-10 after the start of co-culture with MM.1S cells for **(A)** TNF-α, **(B)** IFN-γ, and **(C)** IL-2. Co-cultured cells were incubated at 37°C degrees for 5 hours in the presence of brefeldin **(A)** N=3 separate donors, mean ± SEM. *p<0.05, **p<0.01. **(D)** Combinatorial gating function in Flow Jo software and SPICE analysis were utilized to determine the ability of individual cells to produce multiple cytokines during long-term co-culture with MM.1S cells. Results from T cells from three separate donors are shown in rows. Each pie chart represents one treatment group. Slices within the chart represent fractions of the total T cell population organized based on the number of cytokines, which T cells contained in that fraction produced in response to treatment (white-0, yellow-1, blue-2, red-3). Arcs on the outside of the pie chart signify which cytokine(s) were produced by T cells denoted within the corresponding slice of the pie chart (magenta-IFN-γ, green-TNF-α, orange-IL2), ns-non-specific.

To evaluate the CAR cytokine responses, we compared the ability of armored and non-armored CAR T cells B2ARM and B2, respectively, to produce TNF-α, IFN-γ and IL-2 after long-term co-culture with MM.1S cells (**Figure 5**). Assessment of cytokine production by T cells was carried out by flow cytometric analysis using fix and permeabilize protocol, on day 8-10 of long-term co-culture with MM.1S target cells, in the presence or absence of 10 ng/ml TGF-β. In the presence of TGF-β, there was a statistically significant decrease in the percentage of the non-armored B2 CAR T-cells producing TNF-α, as compared to TGF-β-free culture, p<0.05, (**Figure 5A**). The armored CAR B2ARM resisted the suppression seen with B2 CAR in the presence of TGF-β, although this effect was not statistically significant. IFN-γ production was also strongly suppressed in B2+TGFB CAR group as compared to B2 alone, and partially restored in the B2ARM+TGFB group, however these effects were not statistically significant due to large variability between samples from different donors and experiments (**Figure 5B**). Similarly the suppression of IL-2 production in B2+TGFB experimental group was partially attenuated in the B2ARM+TGFB group, although this effect was also not statistically significant (**Figure 5C**).

Polyfunctional CAR T cell populations, capable of co-expression of more than one cytokine by the same T cell, were associated with greater clinical responses in a clinical trial of CD19 CAR in non-Hodgkin’s lymphoma (45). We therefore evaluated the distribution of polyfunctional CAR T cells in samples shown in **Figures 5A**–**C**. Co-expression of cytokines in individual T cells within each experimental group, as acquired by flow cytometry, was evaluated by combinatorial gating function in Flow Jo software followed by SPICE analysis (46).

Each of the three donors was evaluated for cytokines co-expression after long term co-incubation assay with MM.1S target cells, for CAR B2 and CAR B2ARM, in the presence or absence of TGF-β (**Figure 5D**). Each pie chart represents one treatment group. In the B2 group (without TGF-β), depicted in the first column on the left, more than half of all T cells co-expressed either one (yellow slice), two (blue slice), or three (red slice) cytokines, and slightly less than half T cells expressed no cytokines (white slice). The cytokines expressed singly, or co-expressed by the polyfunctional T cells are IL-2 (orange arc), IFN-γ (magenta arc), and TNF-α (green arc). The armored B2ARM CAR T cells (second column on the left) and the non-armored B2 CAR T cells cultured without TGF-β (first column on the left) showed a similar distribution of polyfunctional T cell fractions when compared for the same donor, and overall had large numbers of cytokine-producing polyfunctional cells. Upon TGF-β treatment of the non-armored B2 T cells (B2+ TGFB, third column from the left), there was a reduction in the fraction of polyfunctional T cells denoted by the blue and the red pie segments. By contrast, in the armored CAR B2ARM group treated with TGF-β (first column from the right), the distribution of polyfunctional cells resembles that of the untreated B2 and B2ARM groups, with large fractions of cells producing one (yellow slice) or two cytokines (blue pie slice), and a small fraction producing three cytokines (red pie slice). Therefore, the armored B2ARM CAR T-cells had overall maintained their polyfunctional status at the end of the long-term co-incubation with MM.1S targets, in the presence of TGF-β.

**Figure 6:**
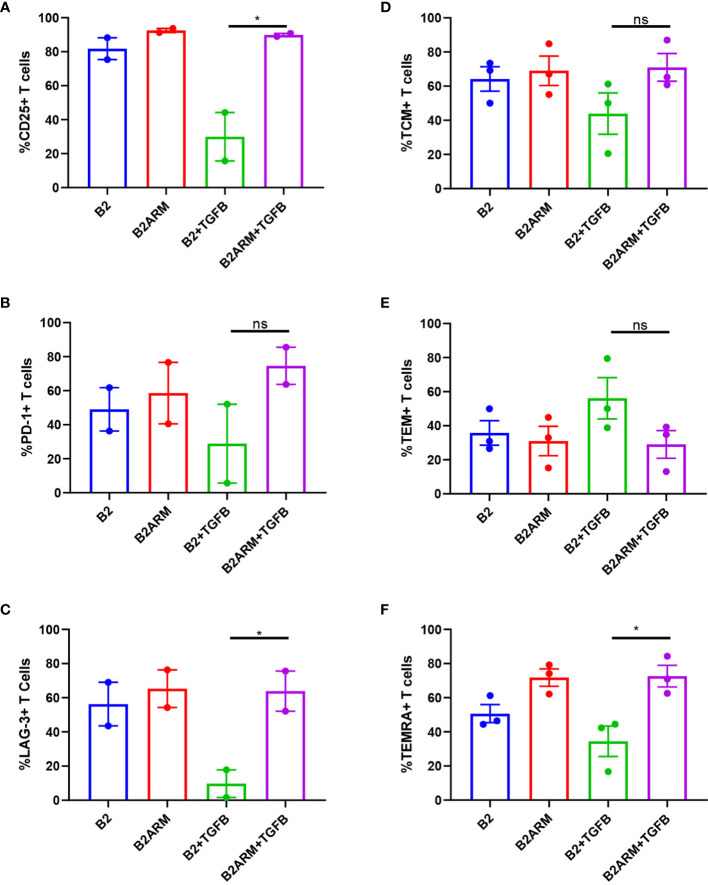
The activation and differentiation capacity of the B2ARM CAR T-cells is not inhibited by TGF-β. Cell surface expression of **(A)** CD25, **(B)** PD-1, **(C)** LAG-3 on CD3+ cells co-cultured with MM.1S were assessed by flow cytometry on day 8 during the second round of co-culture with MM.1S cells in the presence or absence of TGF-β. The percentages of central memory **(D)**, effector memory **(E)**, and TEMRA **(F)** T-cell subsets were determined by the expression of CD62L on CD3+CD45RO+ and CD3+CD45RA cells. Mean values±SEM of 2 to 3 donors from different experiments are shown. Statistically significance was determined Student t-test. *p<0.05, ns-non-specific.

We next examined the phenotypic markers of activation and exhaustion on B2 CAR T-cells and the armored B2ARM CAR T-cells at the conclusion of the course of the long term co-culture with MM cells in the presence of TGF-β. Mean values from separate experiments in two or three donors are presented, from time courses sampled on days 8 or 10. The co-cultured cell populations were gated on live CD3+ T cells, and the percentage of T cell population positive for each marker is shown (**Supplementary Figure 4**). The late activation marker CD25 was decreased on B2 CAR T cells after prolonged exposure to target cells and TGF-β (**Figure 6A**). In contrast, the expression of CD25 on B2ARM CAR T-cells remained similar to CAR T-cells that were not exposed to TGF-β, and was statistically different from the B2+TGFB group (p<0.05, **Figure 6A**). This indicates that the B2ARM CAR was able to maintain its activation status despite the suppressive conditions in the presence of TGF-β. The suppression by TGF-β of the exhaustion markers PD-1 (**Figure 6B**) and LAG-3 (**Figure 6C**), in B2 CARs was also attenuated by the armored B2ARM CAR T, but this effect has not reached statistical significance.

We also assessed the percentages of the specific subsets of memory T-cells, notably central, effector memory, and TEMRA in T cells after co-culture with MM.1S targets in the presence or absence of TGF-β, by looking at the expression of CD62L on CD3+CD45RO+ and CD3+CD45RA+ cells (gating strategy in **Supplementary Figure 5A**). The B2ARM T cells treated with TGF-β attenuated the reduction in the frequency of T effector memory RA-positive T cells (TEMRA) as compared to B2 CAR (p<0.05), but the effects on the central memory (TCM) and effector memory (TEM) subsets were not statistically significant (**Figures 6D–F**). These results underscore the protection of activation and memory differentiation functions in the populations of the armored B2ARM T cells in the presence of TGF-β, as compared to the non-armored B2 CAR.

### The Armored B2ARM CAR Infiltrated Tumors More Efficiently in an *In Vivo* Intradermal Tumor Model as Compared to the B2 CAR

We assessed the functionality of the armored B2ARM CAR in an *in vivo* intradermal xenograft model in NOD.Cg-*Prkdc^scid^ Il2rg^tm1Wjl^*/SzJ (NSG) mice with established tumors of the RPMI-8226 cell line, which produces the latent form of TGF-β (**Supplementary Figure 6A**). We observed a slightly faster decrease in tumor burden of the mice that received the armored B2ARM CAR T-cells compared to B2 CARs, which was not statistically significant (**Supplementary Figure 6B**). However, both CARs were able to completely eradicate tumors, and all mice survived (**Supplementary Figure 6C**). There was no significant difference in weight changes between the armored and the non-armored B2 CAR T-cell treatments (**Supplementary Figure 6D**). We also found a significantly higher percentage of the CD3+CAR+ tumor-infiltrating lymphocytes (TILs), (p=0.04, **Supplementary Figure 6E**), and significantly higher Ki67 staining (p<0.0001, **Supplementary Figure 6F**) in the tumors of mice treated with the armored B2ARM CAR, as compared to the non-armored B2 CAR T-cells, suggesting that the B2ARM CAR T-cells proliferated better at the tumor sites.

**Figure 7:**
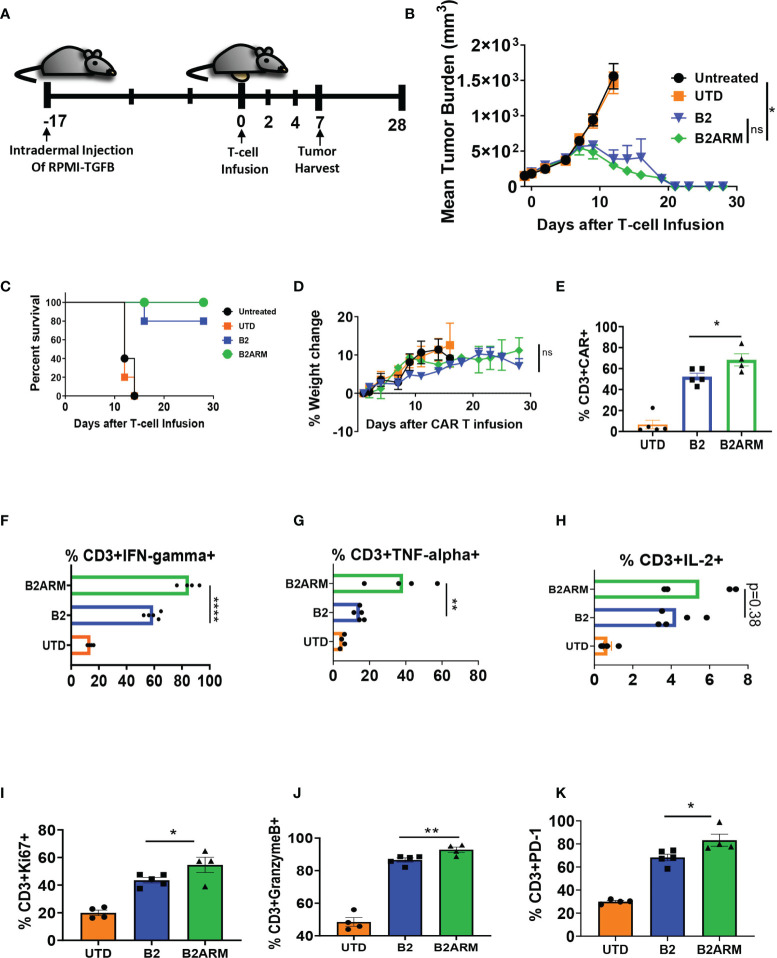
The B2ARM CAR with exhibits superior efficacy *in vivo*. **(A)** NSG mice were intradermally injected on the abdomen with 8 x 10^6^ RPMI-8226 cells with overexpressed TGF-β (n = 10 in all groups except untreated, n = 5). On day 17 after tumor injection, 5 x 10^6^ CAR+ T-cells were intravenously injected. The differences in CAR expression levels were normalized by adjusting the total number of infused T-cells. On day 7 after T-cell infusion, 5 mice from each group (except the untreated group) were sacrificed for tumor harvest, while the rest were monitored for **(B)** tumor progression, **(C)** survival, and **(D)** weight change. **(E)** the percentage of CD3+CAR+ T cells in the tumor homogenates was determined by flow cytometry following tumor harvest. Tumor homogenates were incubated at 37°C for 5 hours in the presence of brefeldin A, and **(F)** IFN-γ, **(G)** TNF-α, and **(H)** IL-2 cytokine production in CD3+ cells was determined by intracellular staining and flow cytometry analysis. Statistical significance was determined by one way ANOVA with Tukey’s *post-hoc* test, *p<0.05, **p<0.01, ****p<0.0001. The percentage of Ki67, **(I)**, Granzyme B, **(J)**, and PD-1, **(K)** expression in CD3+ cells from TIL populations was determined by intracellular or surface staining and flow cytometry in tumor homogenates following tumor harvest. Statistical significance was determined by Student t-test, *p<0.05, **p<0.01, ns, non-specific.

We next evaluated the B2ARM CAR T-cells *in vivo* using the RPMI-TGF-β cell line, which overexpresses the active form of TGF-β (**Figure 7A** and **Supplementary Figure 3B**). Tumors were established for 17 days, and then mice were treated with three million CAR T-positive T cells on study day 0 (**Figure 7A** and **Supplementary Figure 8A**). The armored B2ARM CAR T-cells mediated complete tumor regression in five out of five mice in this model (**Figure 7B**). The non-armored B2 CAR, on the other hand, was only able to clear the tumors in four out of five mice, and the fifth mouse had to be sacrificed due to excessive tumor growth (**Figures 7B, C**). Mice’s weight gain in the course of the study was similar between the non-armored and the armored B2 CAR groups (**Figure 7D**). Tumors were harvested on day 7 after T cell infusion to evaluate the TILs, and the percentage of CD3+CAR+ T cells in the tumors of the mice that received the B2ARM CAR was significantly higher than in the B2 CAR group (**Figure 7E**), despite similar total TIL counts in the tumor (**Supplementary Figure 8B**). In addition, B2ARM TILs elaborated significantly higher IFN-γ (**Figure 7F**) and TNF-α (**Figure 7G**) compared to B2 TILs. The B2ARM TILs similarly had higher IL-2, but the difference did not reach statistical significance, (p=0.38, **Figure 7H**).

We also detected significantly greater degranulation, (%Granzyme B, p<0.05, **Figure 7J**) and activation (%PD-1, p<0.01, **Figure 7K**), as well as higher proliferative capacity (%Ki67+ cells, p<0.05, **Figure 7I**), in B2ARM TILs compared to B2 TILs. Overall, the B2ARM CAR showed functional superiority to B2 CAR in an intradermal RPMI-8226 tumor model with overexpressed TGF-β.

**Figure 8:**
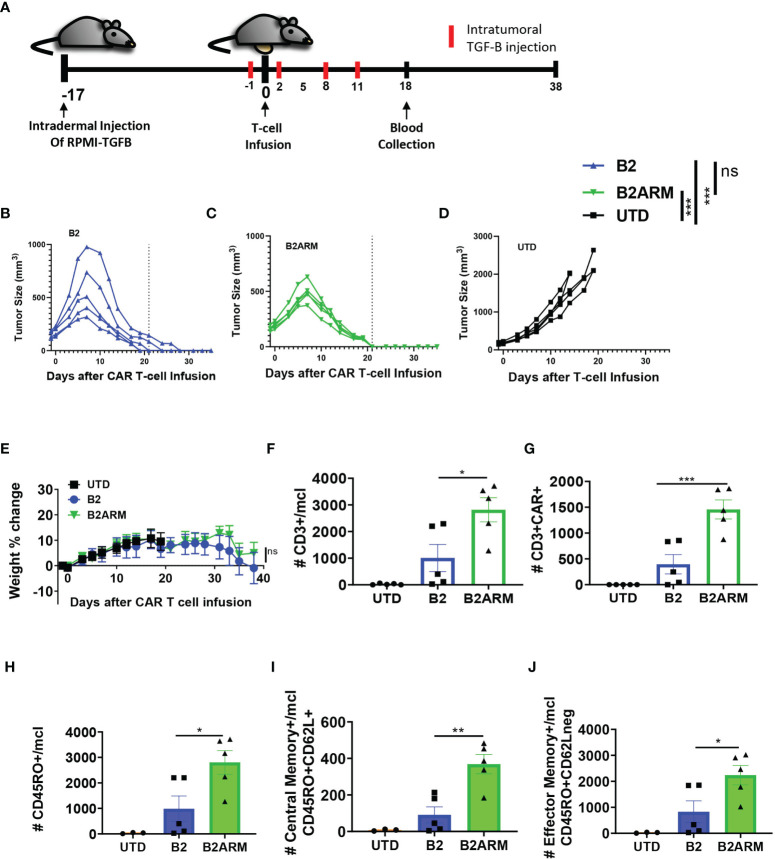
The armored B2ARM CAR demonstrates high anti-tumor efficacy and increased memory T-cell persistence *in vivo*. **(A)** NSG mice were intradermally injected on the abdomen with 8 x10^6^ RPMI-8226 cells with overexpressed TGF-β (n = 5 per group). 2 x 10^6^ CAR+ T-cells were intravenously injected on day 17 after tumor implantation. On the day prior to T-cell infusion (day 16 after tumor implantation), 0.2 ug/ml of TGF-β were intratumorally injected. The exogenous TGF-β treatment was done every 3 days thereafter for a total of 5 injections. The differences in CAR expression levels were normalized by adjusting the total number of infused T-cells. Tumor progression **(B–D)** and changes in weight **(E)** were monitored after T-cell infusion. Dotted line denotes the complete resolution of tumors in the armored CAR T group on day 21, to facilitate comparison between treatments. Changes in tumor size were analyzed by one way ANOVA with Dunnett’s multiple comparisons test, ***p<0.001, ns, non-significant. On day 18 after T cell infusion, the absolute counts of **(F)** CD3+ cells, **(G)** CD3+CAR+ and **(H)** CD45RO+ **(I)** CD45RO+CD62L+, and **(J)** CD45RO+CD62L- cells in the peripheral blood of the mice were determined by counting beads and flow cytometric analysis. Statistical significance was determined by one way ANOVA with Tukey’s multiple comparisons test *p<0.05, **p<0.01.

Having demonstrated highly effective anti-tumor function of the armored BCMA CAR T cells in the xenograft models with or without overexpression of active TGF-β *in vivo*, we proceeded to create an even more challenging set of conditions for the armored CAR T cells. In multiple myeloma, TGF-β production and activation in the bone marrow is achieved through multiple cell types, including the MM cells themselves, as well as the tumor stroma (15, 17–20). To simulate high intratumoral levels of active TGF-β, we created an *in vivo* model combining the intradermal implantation of RPMI8226-TGF-β cell line with repeated intratumoral injection of exogenous active form of TGF-β (**Supplementary Figure 3B**). Additionally, we reduced the treatment dose to only two million CAR T cells per tumor-bearing mouse, as compared to five million per mouse in previous studies (**Figure 8A** and **Supplementary Figure 9A**). Both CAR T groups significantly reduced tumor burden as compared to the UTD control (p<0.001), and the overall magnitude of tumor reduction between B2 and B2ARM CARs was similar. Also, like in our previous *in vivo* studies, we observed fast and complete tumor eradication by day 21 post CAR T treatment in all the tumor-bearing mice that received the armored B2ARM CAR T-cells (**Figure 8B**). In contrast, 2 out of 5 tumor-bearing mice that received the B2 CAR T-cells showed a greater peak tumor burden and slower tumor regression, with only 2 out of 5 mice completely resolving tumors by day 21 (**Figure 8C**). Therefore, both B2 and B2ARM CAR T cells stopped disease progression, however the armored B2ARM CAR achieved complete remissions sooner. Untransduced (UTD) T cells, used as a negative control, did not mediate tumor regression in this model (**Figure 8D**). Weights of mice receiving the armored B2 CAR T-cells remained similar to those receiving the non-armored B2 CAR T-cells throughout the study (**Figure 8E**).

We also examined the expansion and differentiation of the CAR T-cells *in vivo* after tumor exposure in the peripheral blood. On day 18 after CAR T-cell infusion, we observed both significantly higher total human T-cell (CD3+) and CAR T-cell (CD3+CAR+) numbers in the blood of the mice that received the armored B2ARM CAR T-cells compared to the non-armored B2 CARs (**Figures 8F, G**). Furthermore, there were significantly greater numbers of total CD45RO+ cells (**Figure 8H**), central memory (CD45RO+CD62L+, **Figure 8I**) and effector memory (CD45RO+CD62L-, **Figure 8J**) in mice that received the armored B2ARM CAR T-cells, as compared to the non-armored B2 CAR T-cells. These data demonstrate the rapid and effective rejection of the MM tumors by the armored BCMA CAR T-cells, as well as superior expansion and memory differentiation of the armored CAR T cells under the enhanced TGF-β conditions *in vivo*, as compared to the non-armored B2 CAR T-cells.

## Materials and Methods

### Generation of CARs B1, B2 and the TGFBRII Dominant Negative Armored CAR B2ARM

BCMA CARs B1 and B2 were comprised of fully human scFv sequences ScFv1 and scFv2 (**Supplementary Figure 1A**), linked in frame to CD8 hinge and transmembrane domain, 4-1BB/CD137 co-stimulatory domain, and CD3ζ activation domain. In the armored B2ARM construct, the sequence of the extracellular and transmembrane domains of the human TGFBRII (AA 24-192, UniProt ID: A3QNQ0), was cloned downstream of the B2 CAR. The B2 CAR and the TGFBRII were separated by a ribosome skip site (P2A), which was derived from the porcine teschovirus-1 polyprotein (AA 976-997, GenBank ID: CAB40546.1, mutated residue P977S). P2A was flanked on both sides with a furin cleavage site (amino acids: RAKR).

### T-Cell Transduction and Culture

Primary CD4 and CD8 T-cells were activated with TransAct (Miltenyi Biotec, Auburn CA) according to the manufacturer’s protocol. The cells were cultured overnight at a density of 1 x 10^6^ cells/ml in TexMACS media (Miltenyi Biotec, Auburn CA) supplemented with 30 U/ml of recombinant human IL-2 (Miltenyi Biotec, Auburn CA). After 18-24 hours, the T-cells were transduced with lentiviral vectors containing the CAR constructs. The T-cells were incubated with the lentiviral vectors for 2 days, and the cultures were subsequently washed and re-suspended in fresh TexMACS media with IL-2 and maintained at a density of 0.5 x 10^6^ cells/ml. On day 6 or 7 after the start of T-cell culture, the cell surface expression of the CARs was assessed by flow cytometry.

### Flow Cytometry Staining

To assess the cell surface expression of BCMA CARs, 0.5 – 1 x 10^6^ CAR T-cells were resuspended in FACS buffer (Miltenyi Biotec’s AutoMACS Rinsing Solution + MACS BSA Stock Solution) and incubated with 0.5 ug of recombinant human BCMA Fc Chimera Protein (RND Systems, Minneapolis, MN) for 20 mins at 4°C. Cells were washed twice and re-suspended with FACS buffer and incubated for 20 mins at 4°C with anti-Fc-Alexa Fluor 647 at 1 to 200 dilution. Cells were again washed twice and resuspended in FACS buffer and incubated for 20 mins at 4°C with anti-CD4-VioBlue or anti-CD8-VioGreen (Miltenyi Biotec, Auburn CA) at 1:50 dilution and 7AAD at 1:20 dilution. Cells were subsequently washed and analyzed using the MACSQuant Analyzer 10 flow cytometer (Miltenyi Biotec, Auburn CA). The TGF-β receptor II was detected by antibody clone REA903 (Miltenyi Biotec). For exhaustion marker staining, CAR T-cells were resuspended in FACs buffer and incubated with anti-PD-1-PE-Vio 770 (Miltenyi Biotec, Auburn CA) and anti-LAG-3-APC (BioLegend, San Diego, CA) at 1 to 30 dilution. Memory markers were stained by incubating CAR T-cells with anti-CD45RO-PE-Vio 770, anti-CD45RA-APC, and CD62L-PE (Miltenyi Biotec, Auburn CA) at 1 to 30 dilution. For both exhaustion and memory staining panels, cells were additionally stained with CD8-VioGreen, CD3-VioBlue, and 7AAD. Cells were incubated with the antibodies for 20 mins at 4°C, and subsequently washed then acquired using the MACSQuant Analyzer 10 flow cytometer.

For intracellular cytokine staining, T-cells were incubated with target cells for 5-6 hours at 37°C in the presence of Brefeldin A (BD Biosciences, San Jose CA). Cells were subsequently stained as previously described with cell surface markers CD8-VioGreen and CD3-VioBlue. After cell surface staining, cells were fixed and permeabilized with Fixation/Permeabilization Solution Kit (BD Biosciences, San Jose CA) according to the manufacturer’s protocol. Cells were then stained with anti-IFN-γ-APC, anti-TNF-α-APC-Vio 770, and IL-2-PE at dilutions suggested by the manufacturer. Similarly, for additional intracellular T cell marker staining, fixed and permeabilized T cells were stained with antibodies to Granzyme B, or KI67, as indicated. For cell surface staining, non-fixed or permeabilized T cells were stained with antibody to CD107a, or annexin V-FITC binding reagent (Miltenyi Biotec, Cat# 130-097-928). All antibodies were from Miltenyi Biotec (Auburn CA), unless otherwise noted. After staining, cells were analyzed using the MACSQuant Analyzer 10 flow cytometer.

### Long-Term Co-Culture

For the long term co-culture experiment, CAR T-cells were co-cultured with target cells, MM.1S expressing GFP, at an ETT ratio of 0.1 – 0.3. The cells were cultured in 6-well plates with TexMACS media that was either treated with 10 ng/ml of human recombinant TGF-β (Miltenyi Biotec, Auburn CA) or remained untreated. The co-culture was fed by adding TGF-β-treated media or untreated media every 2-3 days. The absolute counts of T-cells and target cells at different time points during the long-term co-culture was assessed by quantifying the number of CD3+ cells and GFP+ cells using flow cytometry. The absolute counts were determined by normalizing the number of acquired cells in a specific volume using CountBright Absolute Counting Beads (Molecular Probes, Eugene OR). When less than 15% of the target cells remained, cells from the co-culture were added to fresh target cells to initiate the subsequent round of co-culture. Additional rounds of co-culture were done until the T-cells no longer proliferated.

### Polyfunctionality Analysis of CAR T Cell Populations

Combinatorial gating function in Flow Jo software and SPICE analysis were utilized to determine the ability of individual cells to produce multiple cytokines during long-term co-culture with MM.1S cells. Each pie chart represents one treatment group. Slices within the chart represent fractions of the total T cell population organized based on the number of cytokines, which T cells contained in that fraction produced in response to treatment (white-0, yelow-1, blue-2, red-3). Arcs on the outside of the pie chart signify which cytokine(s) were produced by T cells denoted within the corresponding slice of the pie chart (magenta-IFN-γ, green-TNF-α, orange-IL2).

### TGF-β ELISA

RPMI-8226 and MM.1S target cell lines were seeded at 25 x 10^3^ or 50 x 10^3^ cells per well in a 96-well plate and cultured for 4 days in TexMACS media. The supernatants were collected and either remained untreated or treated with 1 M HCL for 15 mins to activate latent TGF-β. The Human TGF-β 1 Quantikine ELISA Kit (R&D Systems, Minneapolis MN) was used to detect active TGF-β in the supernatants. The ELISA was performed according to the manufacturer’s protocol.

### *In Vivo* Tumor Model

Female 7 to 8-week old NSG mice (NOD.Cg-*Prkdc^scid^ Il2rg^tm1Wjl^*/SzJ) Stock No: 005557, Jackson Laboratory (Bar Harbor, ME) were intradermally injected on the abdomen with 8 x 10^6^ RPMI-8226 tumor cells. The CAR T-cells were intravenously injected after the tumors were allowed to engraft for 18-20 days and have reached volume sizes of > 60 mm^3^ as measured *via* caliper. Tumor volumes were calculated as: V = (L)*(W^2^)/2, where V-volume, W-tumor width, L- tumor length. For groups receiving CAR T-cells, 5 x 10^6^ - 2 x 10^6^ CAR T-positive cells, normalized per CAR expression percentage, were infused as indicated in each study. The number of T-cells that was infused in the UTD group was the mean of the total T-cells that was injected in the CAR T-cell groups. On day 6-7 after T-cell infusion, where indicated, 3-5 mice from each group (except the untreated group) were sacrificed for tumor harvest, while the rest were monitored for tumor progression and survival. Tumor sizes and body weights were measured every 2-3 days. Mice with tumor sizes reaching > 2000 mm were sacrificed. Where indicated, percentage of body weight change was calculated relative to day 0 of CAR T-cell administration.

## Discussion

The development of various treatment options, including CAR T-cell therapy, has greatly improved the prognosis of MM patients. Nevertheless, most patients eventually relapse, and MM remains an incurable disease. Some of the shortcomings of CAR T-cell therapy in MM have been attributed not only to antigen escape but also to failed expansion and limited persistence of CAR T-cells (4). While the underlying conditions leading to insufficient expansion and persistence of CAR T-cells have yet to be fully elucidated, it is well-established that the BM microenvironment within which MM cells localize is profuse with various factors that induce the growth of MM cells and foster an immunosuppressive niche (10). One such notable factor is TGF-β, a multifunctional cytokine that is heavily enriched in the BM and is a potent T-cell suppressor. In this study, we have designed a novel BCMA CAR that co-expresses the dominant-negative form of TGFB receptor type 2, B2ARM, in order to confer resistance to the CAR T-cells from the suppressive effects of TGF-β that is abundantly deposited in the BM milieu.

The B1 and B2 CAR T cells incorporate fully-human scFv sequences. In comparison to B1, B2 CAR T-cells exhibited high transduction efficiency and potent cytotoxicity in overnight killing assays against target MM cell lines, MM.1S and RPMI-8226. Moreover, the B2 CAR exhibited superior functionality compared to B1 CAR in long-term co-culture with MM.1S cells. B2 CAR retained potent cytotoxic capability despite repeated and prolonged exposure to MM cells, whereas B1 CAR showed reduced killing of target cells after prolonged co-culture with MM cells. The ability of the B2 CAR T- cells to maintain a robust proliferative capacity after prolonged exposure to MM cells is crucial for clinical success, as high expansion of CAR T-cells in the blood has been associated with better clinical responses for patients treated with other BCMA CAR T-cells (7, 9). Moreover, the B2 CAR T-cells elaborated greater IL-2, IFN-γ, and TNF-α compared to B1 during long-term exposure to tumor cells. Accordingly, when evaluated in RPMI-8226 tumor model of MM *in vivo*, B2 CAR T-cells mediated greater tumor rejection as compared to B1 CAR T-cells.

TGF-β plays an important role in the etiology on MM, and the MM bone marrow tumor microenvironment is TGF-β-rich, owing to both production of this cytokine by the MM cells, and by stromal cells, fibroblasts and osteoblasts in the resorpted bone marrow (15–17). In order to protect the B2 CAR against the suppressive bone marrow microenvironment, we created an armored version of the B2 CAR, termed B2ARM, co-expressing the dominant-negative form of TGF-β receptor 2, (DN TGFBRII). The DN TGFBRII retains the capability of the native receptor to bind TGF-β and form a heterocomplex with TGFBRI, but it lacks the kinase domain needed to phosphorylate TGFBRI and initiate the signaling cascade (43). The DN TGFBRII competes with the native TGFBRII for forming a heterocomplex with TGFBRI (43). Previous work utilizing various dominant-negative forms of TGFBRII in conjunction with CARs revealed resistance to TGFβ-mediated suppression in pre-clinical models of prostate cancer and glioblastoma (47, 48). The dominant-negative TGRBRII approach has also been utilized in a clinical trial of EBV- and tumor-antigen-specific T-cells for the treatment of Hodgkin’s lymphoma and resulted in improved T-cell expansion and persistence, with 4 out of 7 patients achieving durable clinical responses (49).

Here, we show that the armored B2ARM CAR T-cells, but not the original B2 CAR T-cells, retained their functional potency in the presence of the suppressive TGF-β. In the long term co-culture with MM cell line MM.1S, the B2ARM CAR T-cells exhibited robust proliferation and cytotoxicity, despite prolonged treatment with exogenous TGF-β. The cytotoxic capability of the B2ARM CAR T-cells during exposure to TGF-β remained similar to its untreated counterpart, while the B2 CAR without DN TGFBRII failed to clear the tumor cells with TGF-β treatment. Taken together, these results demonstrate that the B2ARM CAR T-cells are resistant to TGF-β. Additionally, since TGF-β has been documented to induce apoptosis through the intrinsic and extrinsic pathway (50, 51), we specifically evaluated whether the unaffected expansion of B2ARM CAR T-cells in the presence of TGF-β was also a consequence of resistance to TGF-β induced-apoptosis. We observed that there was no difference in annexin V staining between B2ARM and B2 CAR T-cells after exposure to TGF-β. In contrast, the proliferation marker, Ki67, remained highly expressed in B2ARM CAR T-cells, but considerably decreased in B2 CAR T-cells with TGF-β treatment. Therefore, the B2ARM CAR T-cells maintained their proliferative capacity in the presence of TGF-β and apoptosis did not play a role in this system.

TGF-β is known to suppress the effector functions of T-cells by inhibiting granzyme B and cytokine expression (26, 28). Consistently, we show that the non-armored B2 CAR T-cells had severely decreased granzyme B, CD107a, and TNF-α production after prolonged exposure to TGF-β. By contrast, the expression of these effector molecules in B2ARM CAR T-cells treated with TGF-β remained closely similar to its TGF-β -untreated counterpart, and the non-armored non-TGFβ– treated CAR T group. Moreover, despite the suppressive conditions of TGF-β treatment, B2ARM CAR T-cells largely retained their polyfunctionality, as exhibited by the ability of a single CAR T-cell to produce multiple cytokines. Polyfunctionality is one of the hallmarks of CAR T-cell potency (52), and thus for the B2ARM CAR to retain polyfunctionality under suppressive conditions may confer a clinical advantage. In addition to regulating the expression of effector molecules, TGF-β has also been known to suppress the differentiation of T-cells to effector cells (45). In accordance to this, we show that in the presence of TGF-β, the non-armored B2 CAR T-cells had impaired differentiation to the TEMRA subset, even after continued engagement with MM cells, in contrast to the armored B2ARM CAR T-cells, which differentiated normally to the effector populations in response to MM cells, despite prolonged treatment with TGF-β.

To evaluate the effectiveness of B2ARM CAR T-cells *in vivo*, we used three separate xenograft models of intradermal implantation of tumor cells into NSG mice. In the first model, the parental RPMI-8226 MM cells, expressing the latent form of TGF-β, were implanted. In the second model, a modified RPMI-TGFB cell line, engineered to secrete the active form of TGF-β, was implanted. In a third model, RPMI-TGFB cells were implanted and tumors were injected with the active form of TGF-β every three days for a total of 5 injections in the course of CAR T treatment.

In all three models, including the parental RPMI-8226 tumor cells, and RPMI-TGFB *in vivo* models with or without TGF-β injection, the armored B2ARM CAR T-cells eradicated tumors as well as B2 CAR T-cells. Additionally, the B2ARM CAR T-cells treatment resulted in a modest survival benefit for mice that were implanted with the RPMI-TGFB cell line. In addition, B2ARM CAR T cells were present in tumors in greater numbers as compared to the non-armored B2 CARs, and T cells extracted from tumor tissue of B2ARM CAR T – treated mice demonstrated greater cytokine and granzyme B production, in support of the functional superiority of the B2ARM CAR T-cells *in vivo*.

In the RPMI-TGFB model with the added exogenous injection of TGF-β we observed increased presence of memory T-cell populations following treatment, in the peripheral blood of the mice that received the armored B2ARM CAR T-cells compared to the mice that received the B2 CARs. These findings show the capacity of the armored B2ARM CAR T-cells to differentiate to memory T-cell populations. These data also confirm the *in vitro* observation that the armored B2ARM CAR T-cells are able to rapidly differentiate to memory T-cell populations despite prolonged exposure to TGF-β, while the non-armored B2 CAR T-cells had decreased differentiation capacity.

It should be noted that the MM intradermal models have their limitations in fully replicating the full pathology of MM. In patients, MM cells predominantly localize in the TGF-β-enriched bone marrow, where various cells including bone marrow stromal cells, osteoblasts, and MM cells are all high sources of TGF-β (45). Therefore, the long-term exposure *in vitro* model in the presence of continuously high TGF-β levels may better represent the true TGF-β-suppressive BM tumor microenvironment in the patients. Indeed, under TGF-β-high conditions, we were able to demonstrate the superiority of the armored B2ARM CAR T-cells as compared to the non-armored B2 CAR *in vitro*. Taken together, these results underscore the functional advantage of the TGFβ-armor in the B2ARM CAR T-cells in TGF-β-rich environments.

While the dearth of suitable MM tumor microenvironment models which accurately represent the MM milieu in patients currently remains a limitation for CAR T assessment, future studies will aim to develop ex-vivo or *in vivo* models which better recapitulate the complex TGF-β-rich immunosuppressive microenvironment of the MM BM.

The elevated levels of TGF-β in the BM of MM patients has had consequential repercussions, including immunosuppression and the development of myeloma bone disease (53). While the exact causes for the lack of durable responses in CAR T-cell therapy for the treatment of MM have not been determined, the elevated concentration of TGF-β, a highly potent immunosuppressive cytokine, in BM where MM cells localize has to be considered as a contributing factor. Therefore, we have designed a novel BCMA CAR that is highly resistant to the suppressive effects of TGF-β. The armored B2ARM CAR successfully withstood TGF-β-mediated inhibition, and mediated greater expansion and tumor infiltration, degranulation and target cell killing, cytokine secretion, robust activation, differentiation to memory subsets and effector function, persistence and polyfunctionality in the presence of TGF-β, both *in vitro* and *in vivo*. The armored B2ARM CAR T-cell design may therefore help overcome the limitations of the current BCMA CAR T-cell therapies, and prevail in the suppressive tumor microenvironment of multiple myeloma.

## Data Availability Statement

The original contributions presented in the study are included in the article/**Supplementary Material**. Further inquiries can be directed to the corresponding author.

## Ethics Statement

The animal study was reviewed and approved by LabCorp Drug Development, Ann Arbor, MI. All procedures were conducted in compliance with the applicable laws, regulations, and guidelines of the National Institutes of Health and with the approval of LabCopr Drug Development’s Animal Care and Use Committee. LabCorp Drug Development is an AAALAC accredited facility.

## Author Contributions

DS, LA, BW, BD, and ZZ contributed to conception and design of the manuscript. LA, BW, YX, BV, and DW performed experiments and acquired results. LA, YX, PH, BV, DW, ZZ, and DS analyzed and interpreted results. LA, DS, BW, PH, and YX drafted and revised manuscript. DS, LA, BW, PH, YX, ZZ, BD, and PD critically reviewed manuscript. All authors have approved the final version of the manuscript.

## Conflict of Interest

DS, LA, YX, ZZ, BV, DW, PH, PD are employees of Lentigen Technology, a Miltenyi Biotec Company. BW is an employee of Miltenyi Biotec.

The remaining authors declare that the research was conducted in the absence of any commercial or financial relationships that could be construed as a potential conflict of interest.

## Publisher’s Note

All claims expressed in this article are solely those of the authors and do not necessarily represent those of their affiliated organizations, or those of the publisher, the editors and the reviewers. Any product that may be evaluated in this article, or claim that may be made by its manufacturer, is not guaranteed or endorsed by the publisher.

## Supplementary Material

Supplementary Figure 1
**(A)** Amino acid sequences of scFv1 and scFv2. **(B)** The expression of BCMA on MM.1S (left panel) and RPMI-8226 (right panel) multiple myeloma cell lines, as evaluated by flow cytometry.

Supplementary Figure 2 The cell surface expression of **(A)** CAR, **(B)** CD25 and **(C)** CD69 on CD3+ cells during long-term co-culture with MM.1S cells was determined by flow cytometry. Days of co culture are indicated on the x-axis, and days of target cell addition are indicated by arrows in **(A)**. Mean ± SEM of experiments in T cells from two separate donors is shown. Statistical significance was determined by two way ANOVA with Sidak’s multiple comparisons test, ns, non-significant.

Supplementary Figure 3 Analysis of TGFB secretion from native multiple myeloma cell lines MM.1S and RPMI-8226 **(A)**, or RPMI-TGFB clone of the parental RPMI-8226 cell line, engineered to stably overexpress the activated form of TGF-β **(B)**. MM.1S and RPMI-8226 were cultured for 4 days at 37°C and 5% CO_2_, and supernatants were collected and treated with 1M HCL to activate latent TGF-β, or remained untreated. The presence of active TGF-β in the supernatants was detected by ELISA. Data represent mean +/-SEM of three technical replicates, nd – not detected. Statistical significance was determined by two way ANOVA with Sidak’s multiple comparisons *post-hoc* test, ****p<0.001.

Supplementary Figure 4 Gating strategy for the detection of **(A)** activation marker CD25 and **(B)** exhaustion markers LAG-3 and PD-1 in cell populations following long-term co-culture with target cells. Cell co-cultures containing T cells and target cells were stained for the indicated markers and analyzed by flow cytometry. Cell populations were hierarchically gated on lymphocytes based on scatter, then a singlet gate was applied, live cells gated based on 7AAD exclusion, and gated on CD3. The percentage of CD25-positive cells within the CD3+ T cell population was determined based on the fluorescence minus one (FMO) gate. The exhaustion markers gating strategy was similar, except that the final gating was determined by LAG-3 FMO and PD-1 FMO, respectively.

Supplementary Figure 5 Gating strategy to determine the subset of memory T-cells. TEMRA cells were identified by the absence of CD62L on CD3+CD45RA+ cells. The subset of CD3+CD45RO+ that do not express CD62L was considered to be the effector memory T-cells, while the subset of CD3+CD45RO+ cells that express CD62L were identified as the central memory T-cells.

Supplementary Figure 6 The Armored B2ARM CAR exhibits superior tumor infiltration and similar efficacy to B2 CAR in eradicating MM tumors *in vivo*. **(A)** NSG mice were intradermally injected on the abdomen with 8 x10^6^ RPMI-8226 cells (n = 8 except untreated, n = 5). On day 17 after tumor injection, 5 x10^6^ CAR+ T-cells were intravenously injected. The differences in CAR expression levels were normalized by adjusting the total number of infused T-cells. Differences in tumor size were determined by one way ANOVA with Tukey’s multiple comparisons test, **p<0.01. On day 7 after T-cell infusion, 5 mice from each group (except the untreated group) were sacrificed for tumor harvest, while the rest were monitored for **(B)** tumor progression, **(C)** survival, and **(D)** percentage weight change from day 0 of CAR T-cell treatment. The percentage of CD3+CAR+ **(E)**, and the percentage of CD3+Ki67+ F) cells in tumor homogenates were determined by flow cytometry.

Supplementary Figure 7 The percentages of **(A)** CD45RO+, **(B)** CD45RO+ CD62L+, **(C)** CD45RO+ CD62L- were evaluated by flow cytometry in the peripheral blood of mice on day 18 after CAR T-cell infusion. N=3-5, mean ± SEM. Statistical significance was determined by one way ANOVA with Tukey’s *post-hoc* test.

Supplementary Figure 8 Expression of B2 and B2ARM CAR T cells before and after infusion into mice in study described in main **Figure 7**. **(A)** CAR expression on the transduced T cells was quantified by flow cytometry. Cells were stained with CAR detection reagent, CD8-VioGreen antibody and 7AAD, for the exclusion of dead cells. Cells were gated based on scatter and live gate. CAR positivity gate was set based on the negative control UTD-untransduced T cells. **(B)** Tumors were harvested from mice on day 7 after CAR T treatment, and the absolute counts of CD3+ TIL cells was determined by surface staining and flow cytometry in tumor homogenates. Statistical significance was determined by one way ANOVA with Tukey’s *post-hoc* tests- non-significant.

Supplementary Figure 9 Expression of B2 and B2ARM CAR T cells before and after infusion into mice in study described in main **Figure 8**. **(A)** CAR expression in infused cells in study described in main **Figure 8**. CAR expression on the transduced T cells was quantified by flow cytometry. Cells were stained with CAR detection reagent, CD8-VioGreen antibody and 7AAD, for the exclusion of dead cells. Cells were gated based on scatter and live gate. CAR positivity gate was set based on the negative control UTD-untransduced T cells. **(B)** CAR+ T cell percentage of total human CD3+ T cells in mouse peripheral blood, as determined by flow cytometry. On day 18 after T-cell infusion, the percentage of CAR+ T cells from all CD3+ human T cells in the peripheral blood of the mice was determined by flow cytometric analysis. Statistical significance was determined by unpaired Student t-test. ns, non significant.
