# Supplementary figures and images for: Armored BCMA CAR T Cells Eliminate Multiple Myeloma and Are Resistant to the Suppressive Effects of TGF-β
Source: Front Immunol. 2022 Feb 9;13:832645. doi: 10.3389/fimmu.2022.832645 (PMC8863610; doi:10.3389/fimmu.2022.832645)

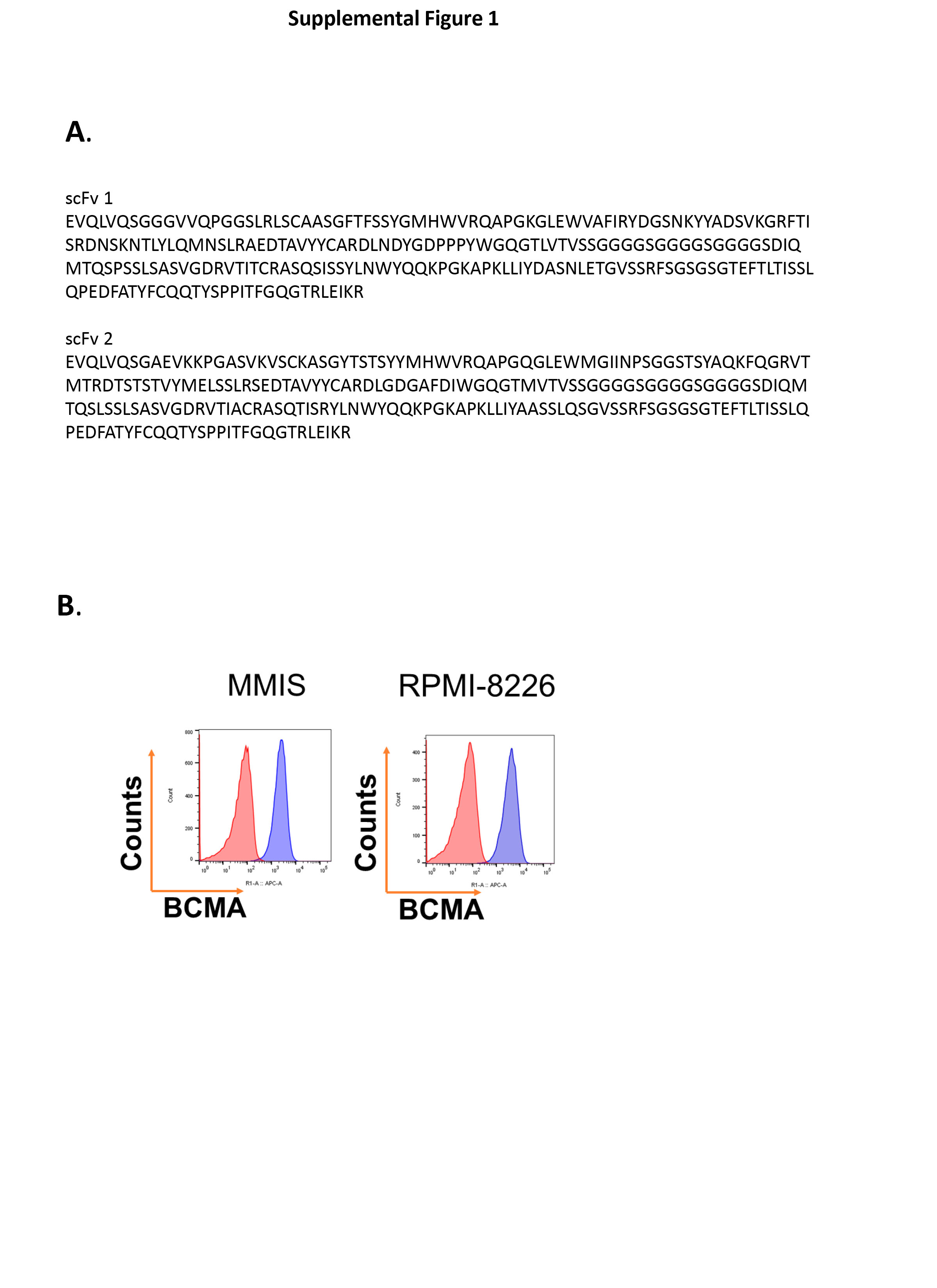

Supplement: Supplementary Figure 1 — (A) Amino acid sequences of scFv1 and scFv2. (B) The expression of BCMA on MM.1S (left panel) and RPMI-8226 (right panel) multiple myeloma cell lines, as evaluated by flow cytometry. [file Image_1.jpeg]

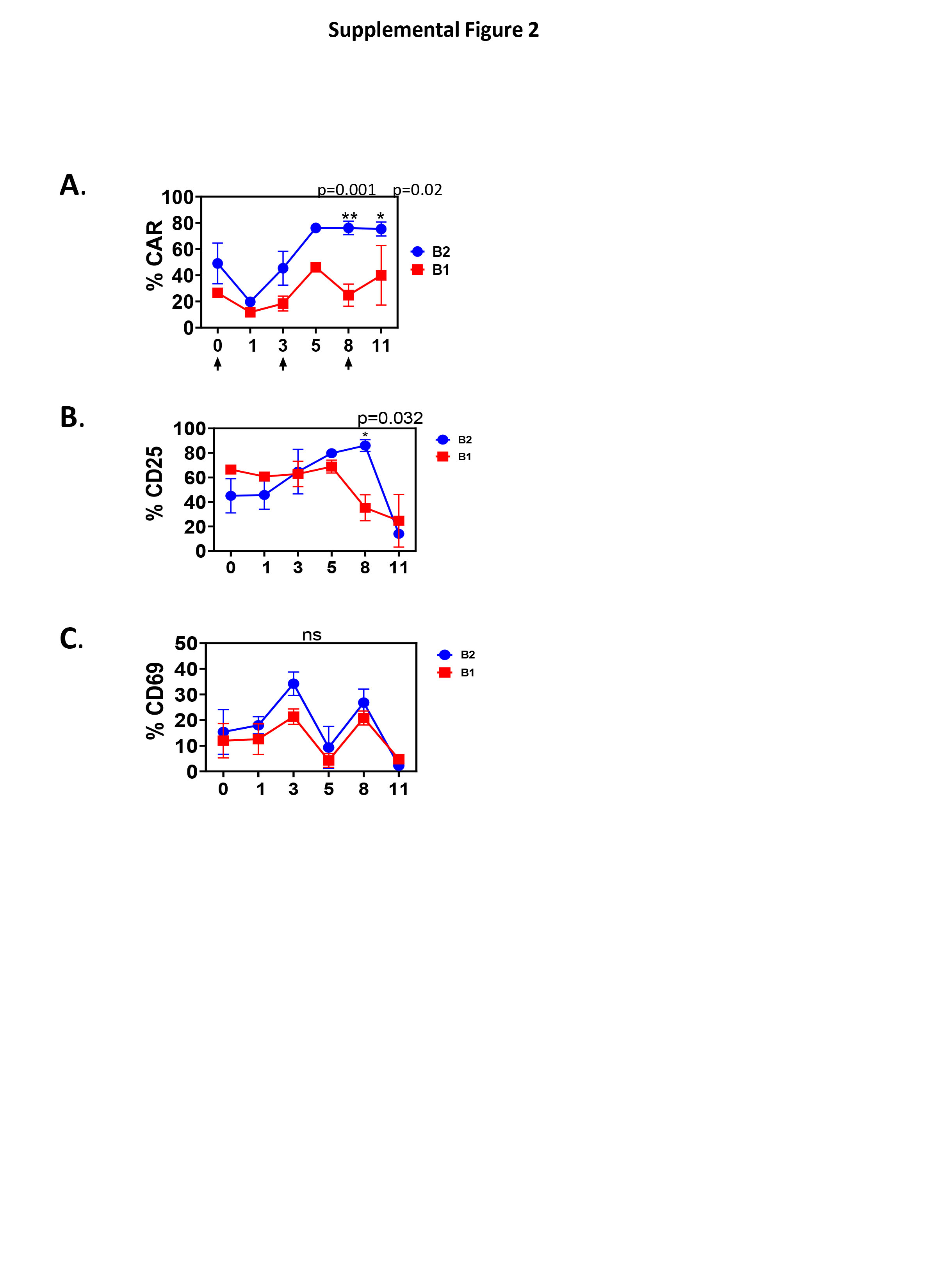

Supplement: Supplementary Figure 2 — The cell surface expression of (A) CAR, (B) CD25 and (C) CD69 on CD3+ cells during long-term co-culture with MM.1S cells was determined by flow cytometry. Days of co culture are indicated on the x-axis, and days of target cell addition are indicated by arrows in (A). Mean ± SEM of experiments in T cells from two separate donors is shown. Statistical significance was determined by two way ANOVA with Sidak’s multiple comparisons test, ns, non-significant. [file Image_2.jpeg]

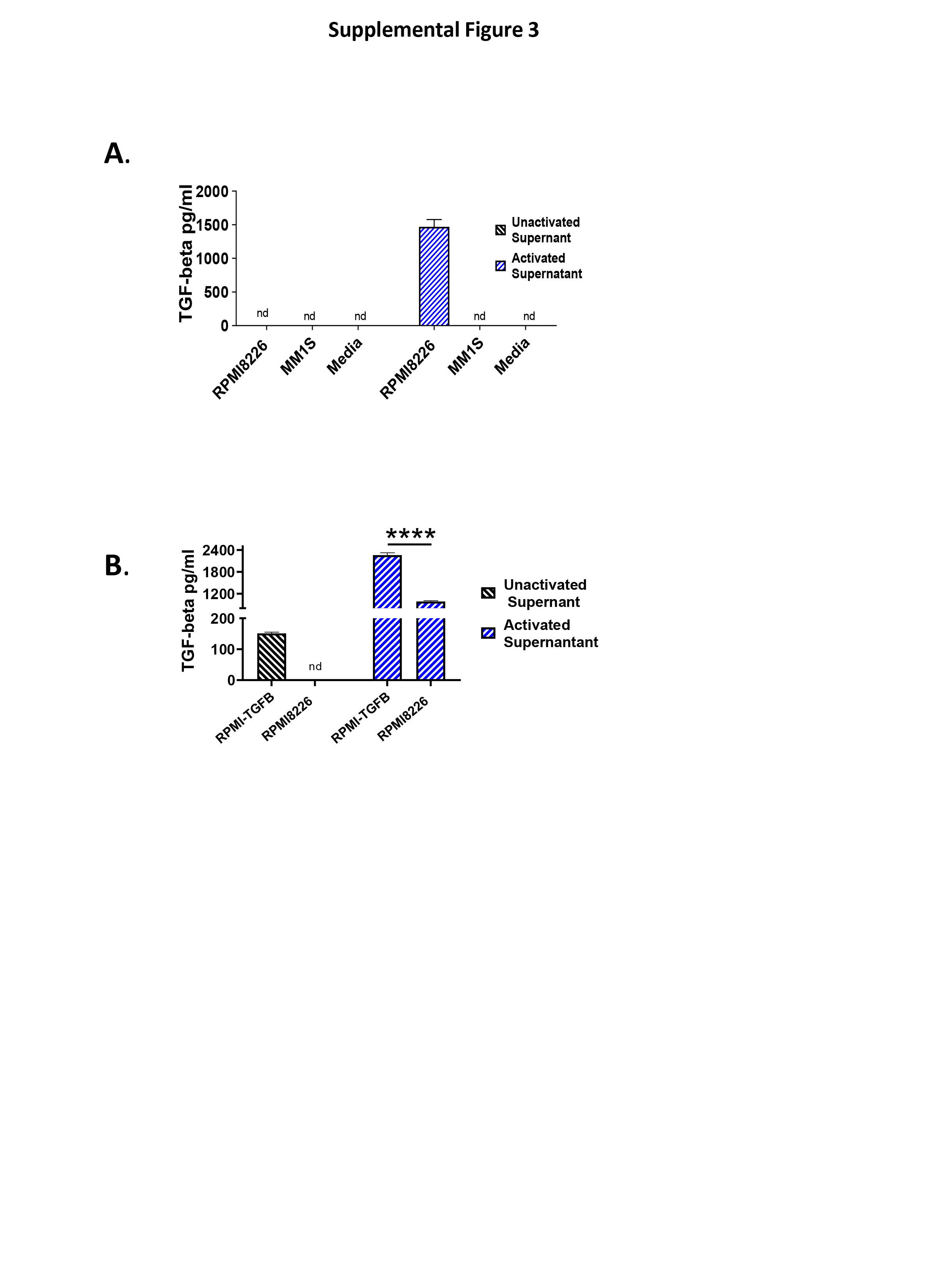

Supplement: Supplementary Figure 3 — Analysis of TGFB secretion from native multiple myeloma cell lines MM.1S and RPMI-8226 (A), or RPMI-TGFB clone of the parental RPMI-8226 cell line, engineered to stably overexpress the activated form of TGF-β (B). MM.1S and RPMI-8226 were cultured for 4 days at 37°C and 5% CO2, and supernatants were collected and treated with 1M HCL to activate latent TGF-β, or remained untreated. The presence of active TGF-β in the supernatants was detected by ELISA. Data represent mean +/-SEM of three technical replicates, nd – not detected. Statistical significance was determined by two way ANOVA with Sidak’s multiple comparisons post-hoc test, ****p<0.001. [file Image_3.jpeg]

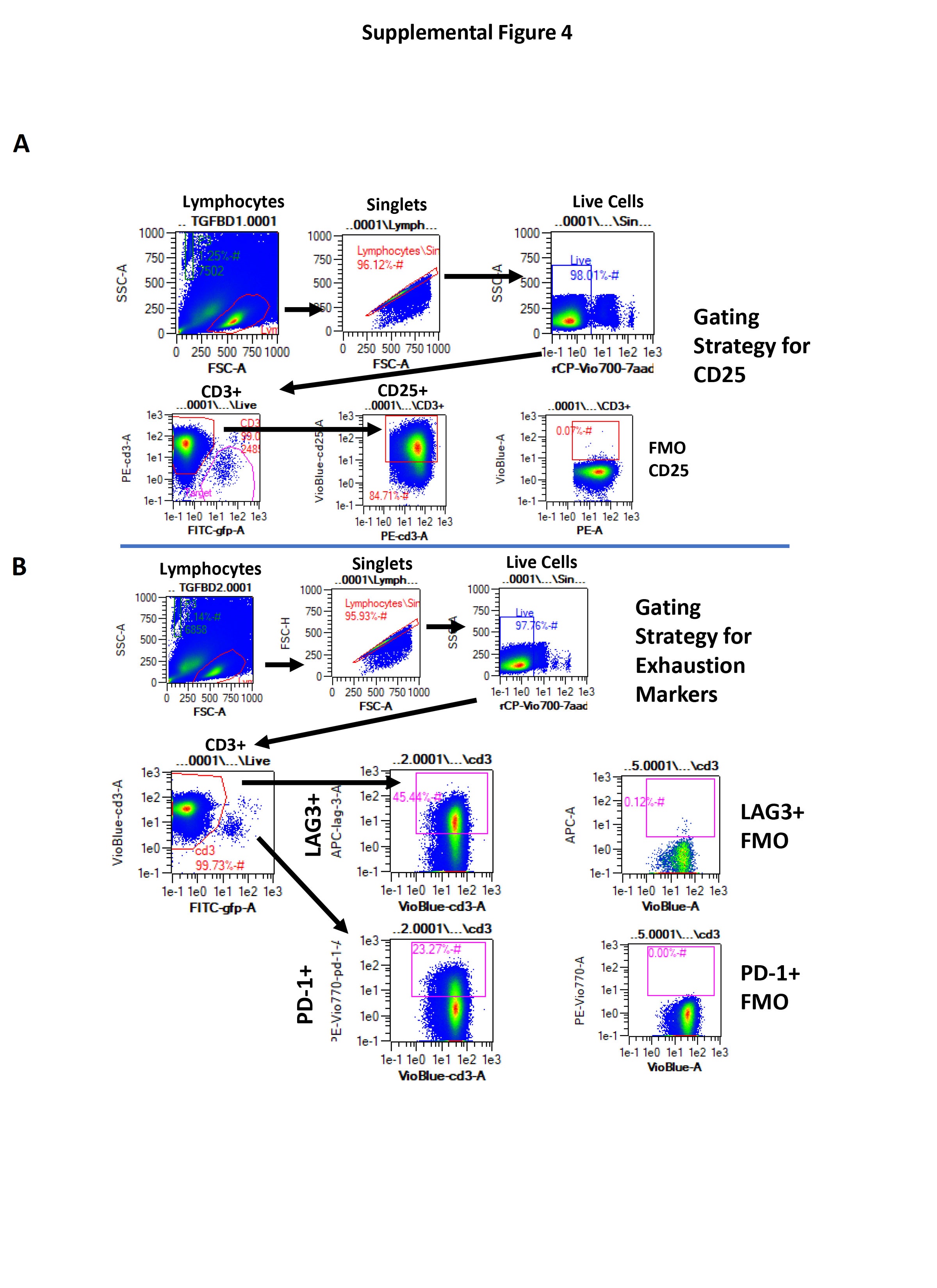

Supplement: Supplementary Figure 4 — Gating strategy for the detection of (A) activation marker CD25 and (B) exhaustion markers LAG-3 and PD-1 in cell populations following long-term co-culture with target cells. Cell co-cultures containing T cells and target cells were stained for the indicated markers and analyzed by flow cytometry. Cell populations were hierarchically gated on lymphocytes based on scatter, then a singlet gate was applied, live cells gated based on 7AAD exclusion, and gated on CD3. The percentage of CD25-positive cells within the CD3+ T cell population was determined based on the fluorescence minus one (FMO) gate. The exhaustion markers gating strategy was similar, except that the final gating was determined by LAG-3 FMO and PD-1 FMO, respectively. [file Image_4.jpg]

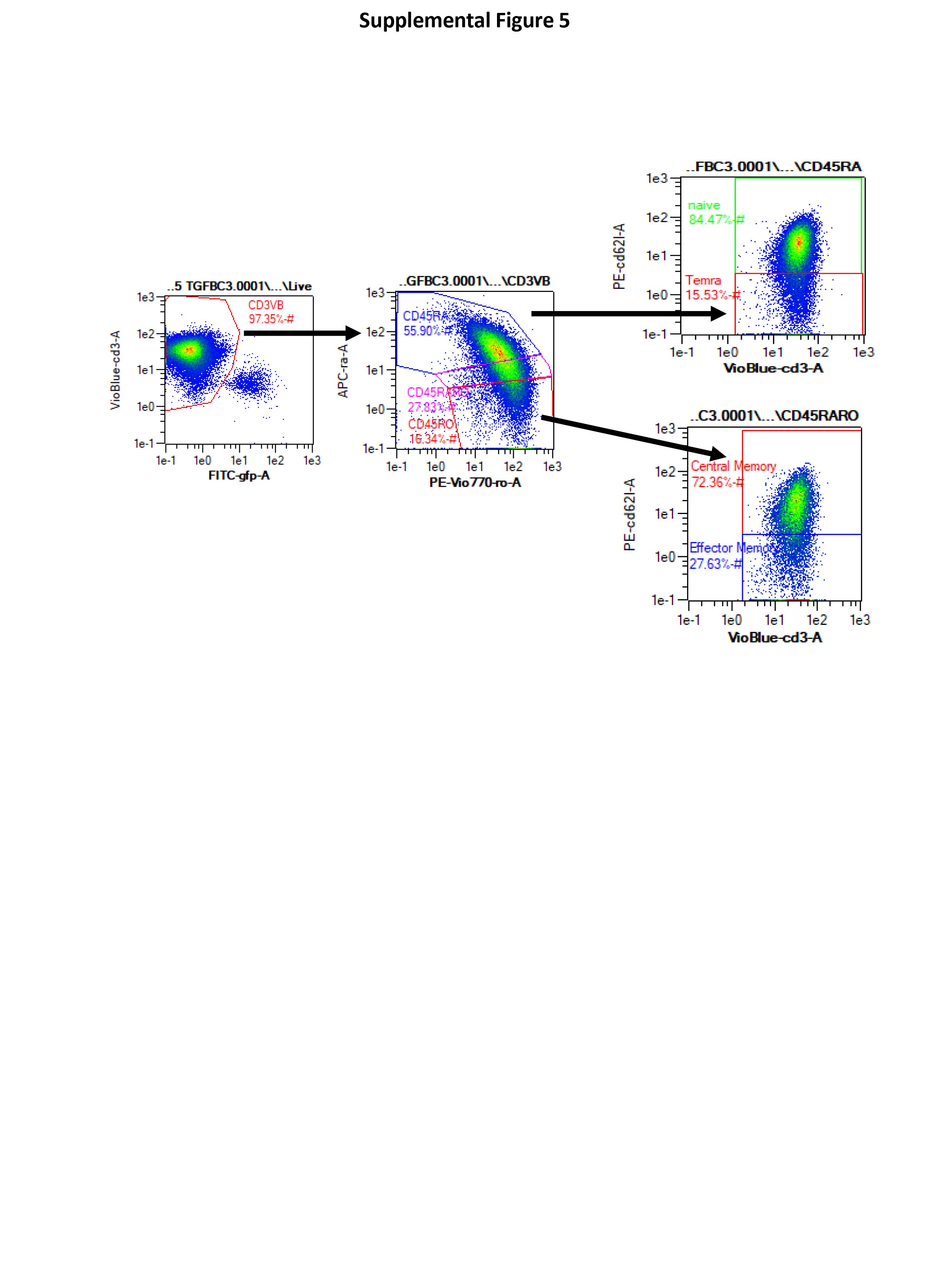

Supplement: Supplementary Figure 5 — Gating strategy to determine the subset of memory T-cells. TEMRA cells were identified by the absence of CD62L on CD3+CD45RA+ cells. The subset of CD3+CD45RO+ that do not express CD62L was considered to be the effector memory T-cells, while the subset of CD3+CD45RO+ cells that express CD62L were identified as the central memory T-cells. [file Image_5.jpg]

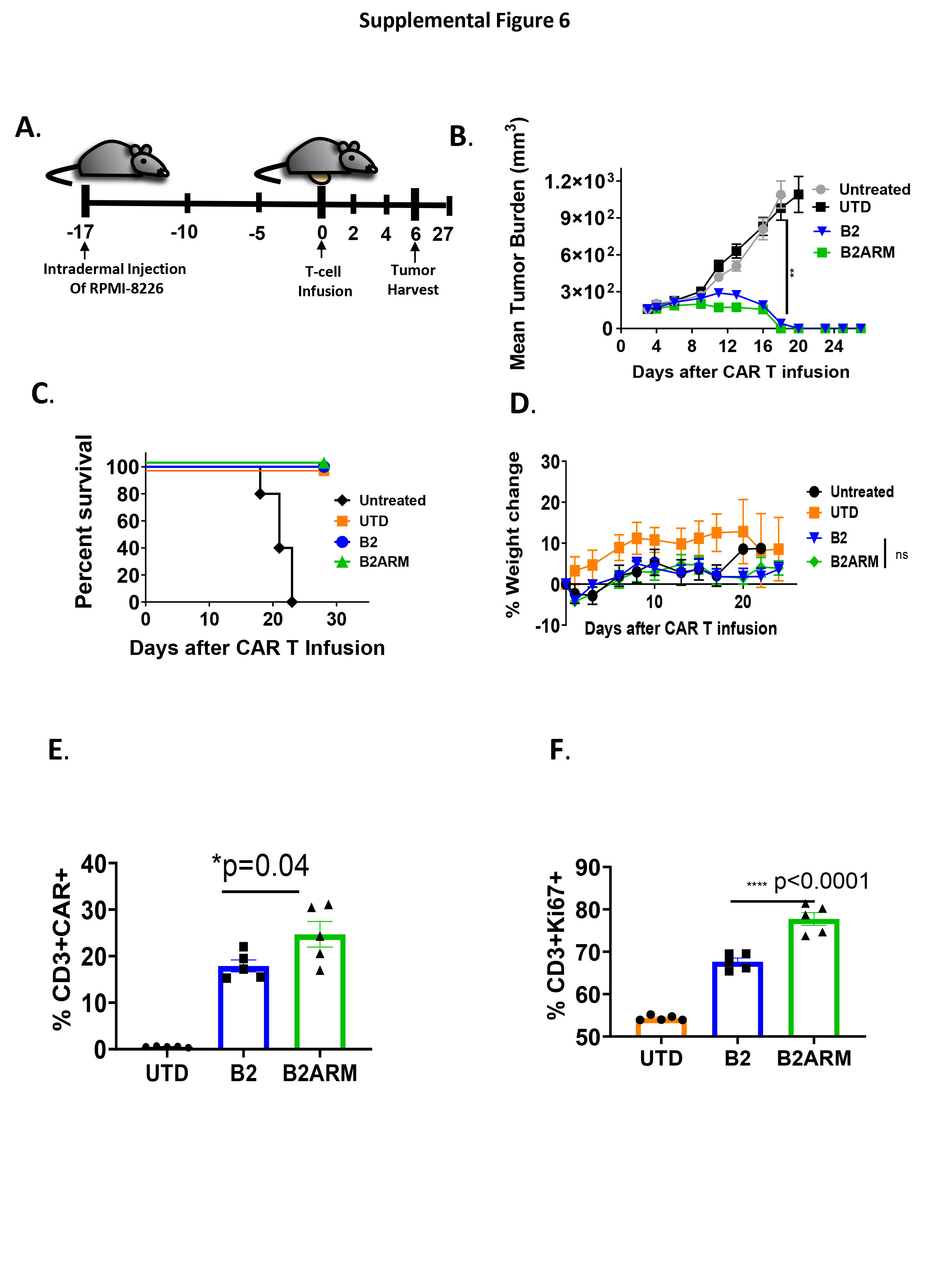

Supplement: Supplementary Figure 6 — The Armored B2ARM CAR exhibits superior tumor infiltration and similar efficacy to B2 CAR in eradicating MM tumors in vivo. (A) NSG mice were intradermally injected on the abdomen with 8 x10^6^ RPMI-8226 cells (n = 8 except untreated, n = 5). On day 17 after tumor injection, 5 x10^6^ CAR+ T-cells were intravenously injected. The differences in CAR expression levels were normalized by adjusting the total number of infused T-cells. Differences in tumor size were determined by one way ANOVA with Tukey’s multiple comparisons test, **p<0.01. On day 7 after T-cell infusion, 5 mice from each group (except the untreated group) were sacrificed for tumor harvest, while the rest were monitored for (B) tumor progression, (C) survival, and (D) percentage weight change from day 0 of CAR T-cell treatment. The percentage of CD3+CAR+ (E), and the percentage of CD3+Ki67+ F) cells in tumor homogenates were determined by flow cytometry. [file Image_6.jpeg]

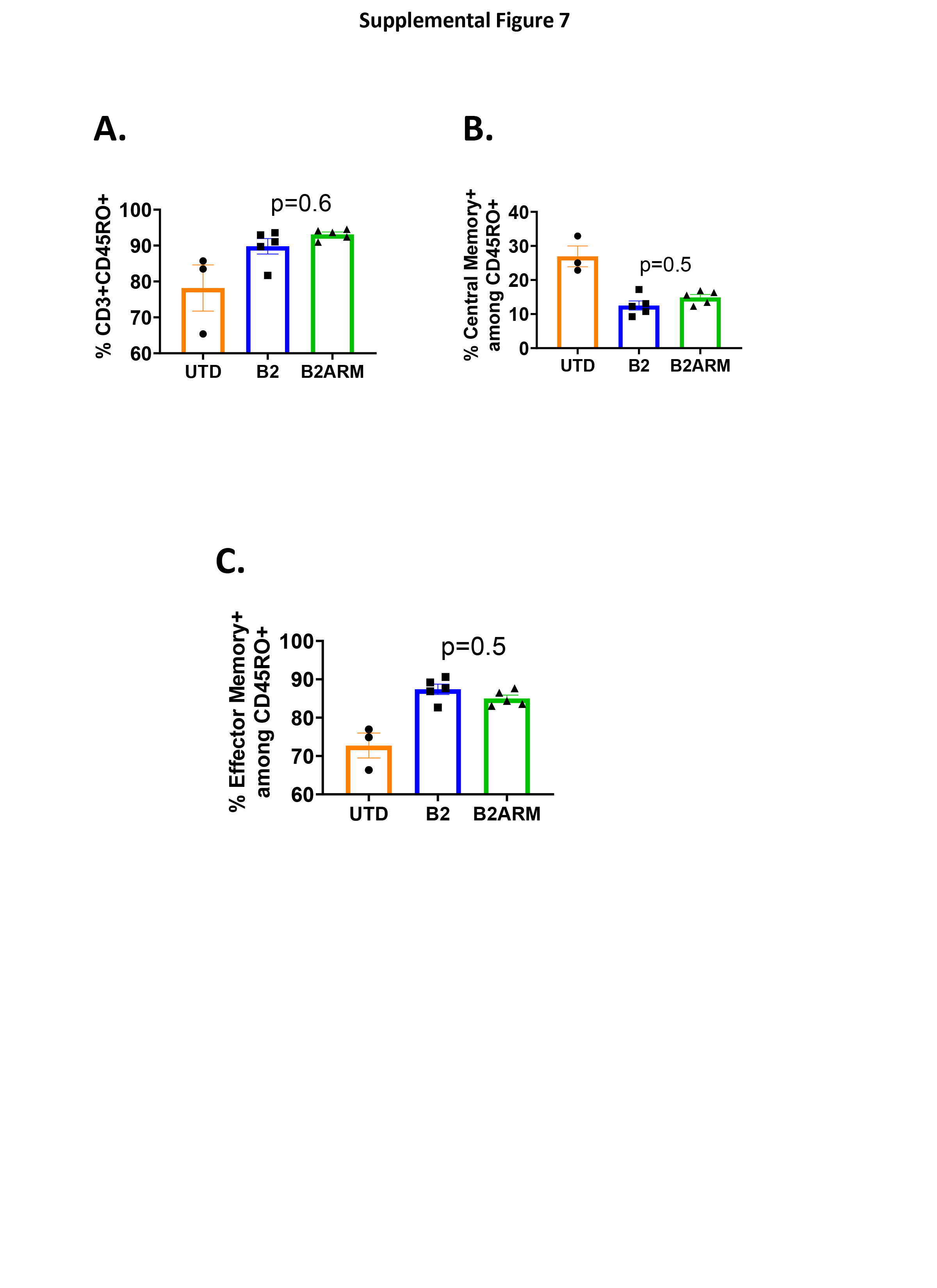

Supplement: Supplementary Figure 7 — The percentages of (A) CD45RO+, (B) CD45RO+ CD62L+, (C) CD45RO+ CD62L- were evaluated by flow cytometry in the peripheral blood of mice on day 18 after CAR T-cell infusion. N=3-5, mean ± SEM. Statistical significance was determined by one way ANOVA with Tukey’s post-hoc test. [file Image_7.jpeg]

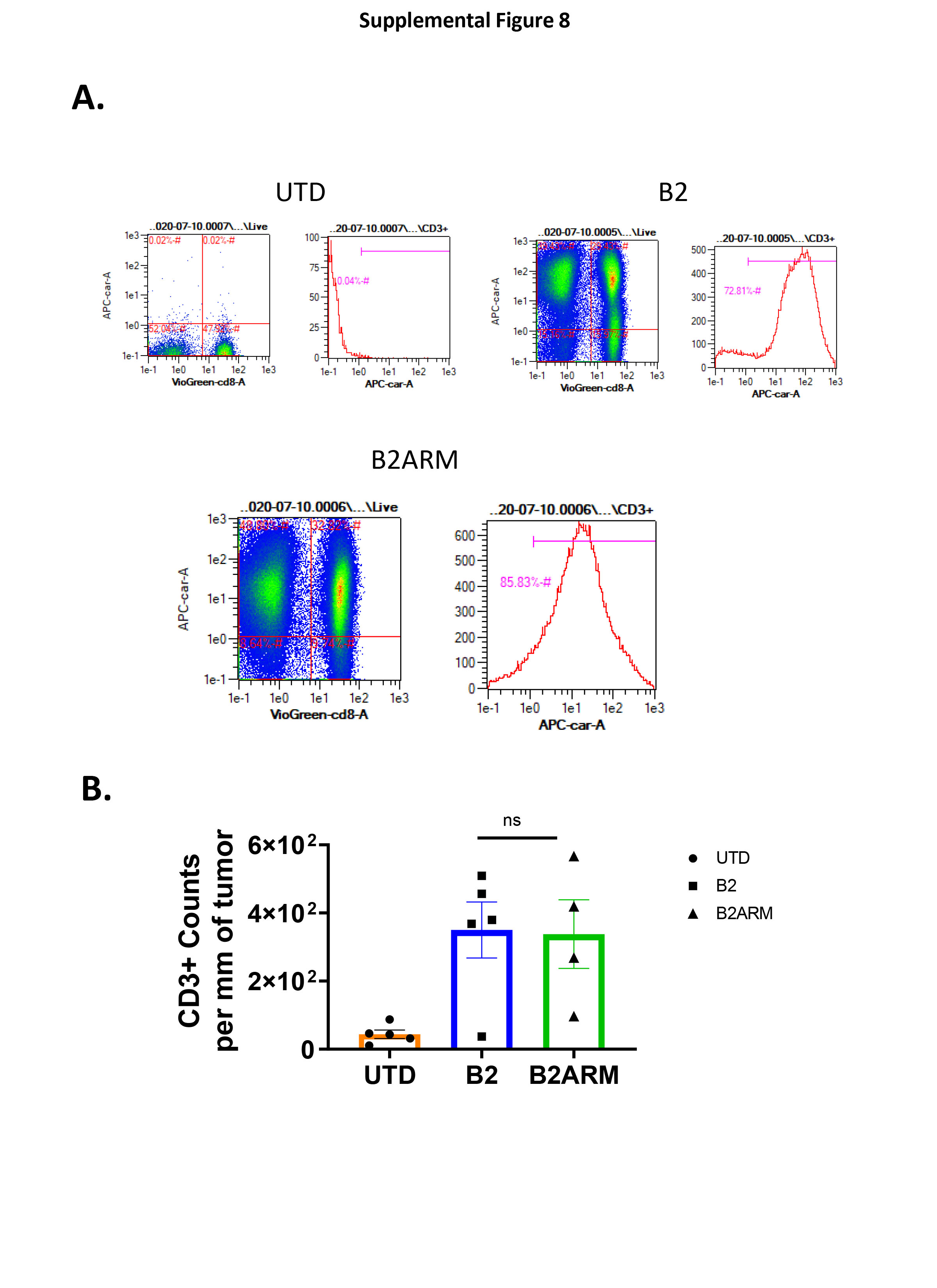

Supplement: Supplementary Figure 8 — Expression of B2 and B2ARM CAR T cells before and after infusion into mice in study described in main Figure 7 . (A) CAR expression on the transduced T cells was quantified by flow cytometry. Cells were stained with CAR detection reagent, CD8-VioGreen antibody and 7AAD, for the exclusion of dead cells. Cells were gated based on scatter and live gate. CAR positivity gate was set based on the negative control UTD-untransduced T cells. (B) Tumors were harvested from mice on day 7 after CAR T treatment, and the absolute counts of CD3+ TIL cells was determined by surface staining and flow cytometry in tumor homogenates. Statistical significance was determined by one way ANOVA with Tukey’s post-hoc tests- non-significant. [file Image_8.jpeg]

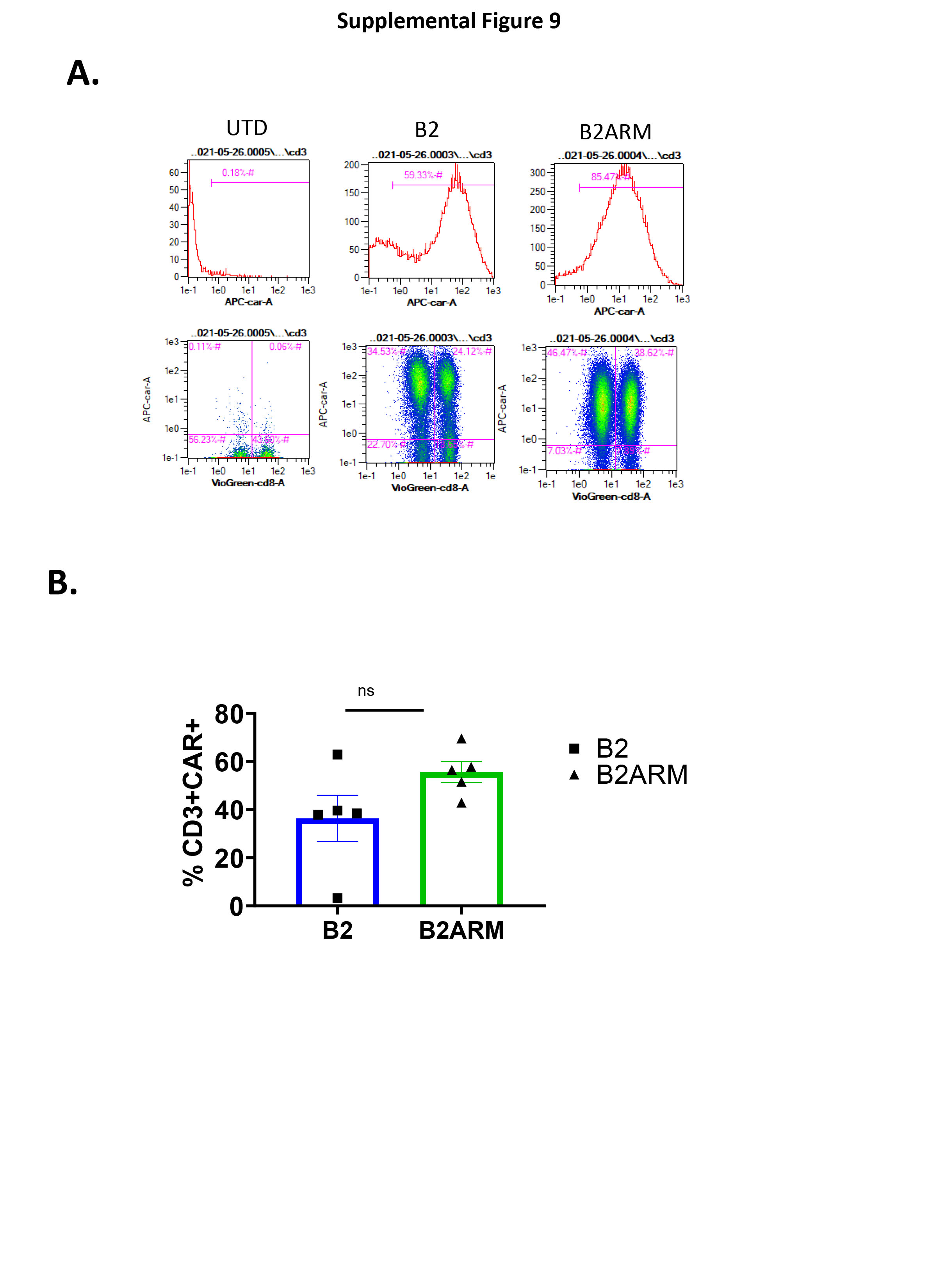

Supplement: Supplementary Figure 9 — Expression of B2 and B2ARM CAR T cells before and after infusion into mice in study described in main Figure 8 . (A) CAR expression in infused cells in study described in main Figure 8 . CAR expression on the transduced T cells was quantified by flow cytometry. Cells were stained with CAR detection reagent, CD8-VioGreen antibody and 7AAD, for the exclusion of dead cells. Cells were gated based on scatter and live gate. CAR positivity gate was set based on the negative control UTD-untransduced T cells. (B) CAR+ T cell percentage of total human CD3+ T cells in mouse peripheral blood, as determined by flow cytometry. On day 18 after T-cell infusion, the percentage of CAR+ T cells from all CD3+ human T cells in the peripheral blood of the mice was determined by flow cytometric analysis. Statistical significance was determined by unpaired Student t-test. ns, non significant. [file Image_9.jpeg]
